# Intravitreal exposure to the SARS-CoV-2 spike protein is associated with aggravated retinal lesions in a murine model of central retinal vein occlusion

**DOI:** 10.3389/fmed.2026.1794421

**Published:** 2026-09-03

**Authors:** Siyi Liu, Zhihui Zhang, Yumin Zhang, Xinglong Wang, Yibin Yu, Yunshan Cao, Yan Zhang

**Affiliations:** 1 Tianjin Key Laboratory of Retinal Functions and Diseases, Tianjin Branch of National Clinical Research Center for Ocular Disease, Eye Institute and School of Optometry, Tianjin Medical University Eye Hospital, Tianjin, China; 2 Heart, Lung and Vessels Center, Sichuan Provincial People's Hospital, University of Electronic Science and Technology of China, Chengdu, Sichuan, China

**Keywords:** central retinal vein occlusion, lengsin, Müller cells, SARS-CoV-2, single-cell RNA sequencing, spike protein

## Abstract

**Purpose:**

To explore effects of SARS-CoV-2 ocular infection on severity of central retinal vein occlusion (CRVO) and the associated cellular and molecular clues.

**Methods:**

The spike protein (SP) was intravitreally administered to mimic the pathological process of SARS-CoV-2 ocular infection. Eight-week-old C57BL/6 male mice were randomly divided into 5 groups: normal control (NOR), CRVO, 0.2 SP + CRVO, 0.4 SP + CRVO, and 0.8 SP + CRVO groups. The CRVO group was intravitreally injected with phosphate-buffered saline, and the SP + CRVO groups were intravitreally injected with full-length recombinant SP of Omicron (B.1.1.529) SARS-CoV-2 at 0.2, 0.4, and 0.8 µg. Two days later, a CRVO model was induced via laser photocoagulation following Rose Bengal injection. Then fluorescein fundus angiography (FFA), optical coherence tomography (OCT), and electroretinogram (ERG) were conducted. The retinas from the CRVO and 0.8 SP + CRVO groups were collected on day 9 after modeling for 10× Genomics single-cell RNA (scRNA) sequencing, and the results were validated by immunofluorescence staining of mouse retinas and SP stimulation of a human retinal Müller cell line (MIO-M1).

**Results:**

In CRVO retinas, FFAs demonstrated reduced intravascular fluorescence dye filling and increased fluorescence dye leakage compared with normal counterparts; OCT revealed edema. Reperfusion was delayed, and the intensity and area of leakage, edema, and electrophysiological functions were significantly aggravated in CRVO retinas pretreated with high-dose SP. ScRNA sequencing revealed dramatically upregulated expression of the Lengsin (*Lgsn*) gene in a Müller cell cluster with a substantially reduced proportion in the CRVO group with SP pretreatment compared with the CRVO group. The gene expression patterns were validated in mouse retinas and MIO-M1 cells. The functional consequences of SP exposure were explored in MIO-M1 cells.

**Conclusions:**

Intravitreal exposure to SP, the major pathogenic factor of SARS-CoV-2, was associated with delayed vessel reperfusion and exacerbated vascular leakage, edema, and electrophysiological functions in CRVO retinas. ScRNA sequencing revealed that *LGSN* upregulation in a subpopulation of retinal Müller cells was associated with SP exposure and aggravated retinal lesions in the context of CRVO. These findings may provide molecular and cellular clues for managing SARS-CoV-2-infection-associated CRVO.

## 1 Introduction

Retinal vein occlusion is the second most common retinal vascular disorder (1). According to the location of the blockade, retinal vein occlusion can be categorized into central retinal vein occlusion (CRVO) and branch retinal vein occlusion (2). Despite its lower incidence, CRVO has more severe outcomes, such as intraretinal hemorrhage, retinal vascular leakage, and retinal edema, resulting in partial or complete vision loss (3). The risk for CRVO increases with age and accompanying cardiovascular conditions, such as systemic arterial hypertension, atherosclerosis, and diabetes. Moreover, dyslipidemia, cigarette smoking, and renal diseases have been associated with an increased incidence of CRVO (4).

Coronavirus disease 2019 (COVID-19) is a respiratory viral infection caused by severe acute respiratory syndrome coronavirus type 2 (SARS-CoV-2). Although COVID-19 is a respiratory disease, it increases the risk of venous and arterial thromboembolism, resulting in systemic thrombotic events, such as deep vein thrombosis and pulmonary thrombosis (5, 6). For instance, studies have revealed that infection with SARS-CoV-2 viral particles involving the full-length spike protein (SP), the major pathogenic factor of SARS-CoV-2, is a risk factor for developing pulmonary embolism (7, 8). Moreover, numerous case reports have demonstrated the occurrence of CRVO following SARS-CoV-2 infection even in patients without any systemic risk factors (9–14), suggesting ocular tropism of SARS-CoV-2 and a role for its SP in causing thromboembolism in retinal veins. According to the results of clinical studies, SARS-CoV-2 may invade the retina through several distinct routes. First, although the detection frequency of SARS-CoV-2 mRNA varies in tear or conjunctiva swabs from COVID-19 patients (15–18), it is generally recognized that, for COVID-19 patients with conjunctivitis in particular, virus-laden aerosols can initially infect ocular surface tissues enriched with SARS-CoV-2 receptors, such as angiotensin-converting enzyme 2 (ACE2) and transmembrane serine protease 2 (TMPRSS2) (19, 20), and then spread to adjacent ocular structures and finally reach the retina (15). Alternatively, after infecting the ocular surface, SARS-CoV-2 viral particles may enter retinal circulation through the mediation of ACE2 and CD147 located on the endothelial cell membranes of ocular capillaries and choroid plexus (21) and then cross the disrupted blood‒retinal barrier and infect the neuroretina via ACE2 and TMPRSS2 receptors on retinal cells (22, 23). In addition, retrograde axonal transport along the trigeminal nerve and optic nerve constitutes a neuroinvasive route (24, 25). Therefore, intravitreal administration of SARS-CoV-2 SP, the major pathogenic protein that facilitates viral entry and elicits inflammatory responses, would be a viable approach for studying the effects of intravitreal exposure to viral SP on the progression and severity of CRVO. Furthermore, the cellular and molecular mechanistic clues associated with such effects remain unknown. The retina is a hierarchical structure composed of distinct cell types. Single-cell RNA (scRNA) sequencing can be leveraged to reveal cell type-specific transcriptomic changes, thereby providing insights into the pathogenesis of retinal diseases at the cellular and molecular levels (26).

Therefore, this study employed full-length recombinant SP of the B.1.1.529 Omicron variant of SARS-CoV-2 and explored the effects of the SP on retinal lesions in a mouse model of CRVO established via intravenous injection of Rose Bengal followed by laser photocoagulation. Furthermore, the cellular and molecular clues associated with these effects were explored via scRNA sequencing and bioinformatics analysis, the results of which were validated via immunofluorescence (IF) in mouse whole mount retinas and retinal sections as well as via real-time quantitative PCR and functional assays in human retinal Müller cells under SP stimulation. The findings in this study provide clues for aggravated retinal lesions associated with intravitreal exposure to SP in the context of CRVO.

## 2 Methods and materials

### 2.1 Animals

Eight-week-old C57BL/6 male mice were purchased from Beijing Vital River Laboratory Animal Technology Co., Ltd. (Beijing, China). The sample size was calculated via the formula *N* = (*Z_α_*+*Z_β_*)^2^ × 2 × (*σ*^2^/*δ*^2^) (27). The statistical power is 0.8, and the 2-sided significance level is 0.05. Under a normal distribution, *α* = 0.05, *Z_α_* = 1.96; 1-*β* = 0.8, *Z_β_* = 0.84. In accordance with prior studies (28, 29), *σ* = 13.01, *δ* = 27.26, and *N* = 3.57. Therefore, at least 3 mice per group were used in the experiments of this study. The mice were raised in a specific pathogen-free grade animal facility at Tianjin Medical University Eye Hospital. They were raised at an ambient temperature of 22–26 °C with a 12-h light/12-h dark cycle and had free access to food and water. All the experimental procedures conformed to the Guide for the Care and Use of Laboratory Animals published by the US National Institutes of Health and were approved by the Laboratory Animal Care and Use Committee of Tianjin Medical University (permission number: SYXK2018-0004).

### 2.2 Intravitreal injection of SARS-CoV-2 recombinant SP

The recombinant SP of Omicron (B.1.1.529) SARS-CoV-2 (Cat No. 11060-CV) was purchased from R&D Systems (Minneapolis, MN, USA). Mouse-adapted SARS-CoV-2, which harbors mutations similar to those of the recombinant SP, has been shown to recapitulate most human symptoms of COVID-19, such as damage to and inflammatory cell infiltration in the lung and viral replication in the respiratory tract, following intranasal inoculation at a dose of 1 × 10^6^ plaque-forming units (PFUs) in mice (30). For SARS-CoV-2, one PFU comprises approximately 1 × 10^4^ virions on average (31, 32), and cryo-electron microscopy has shown that each virion contains up to 50 SP trimers (33), and the molecular weight of the trimeric SP is about 200 kDa; therefore, SARS-CoV-2 at 1 × 10^6^ PFUs contains approximately 0.2 μg of SP. Considering viral replication *in vivo*, 0.2, 0.4, and 0.8 µg of SP would be intravitreally administered to study the effects of SP exposure during COVID-19 on retinal lesions in a mouse CRVO model. Therefore, the recombinant SP was diluted with sterile phosphate-buffered saline (PBS, pH 7.4; Solarbio, Beijing, China) to concentrations of 0.2, 0.4, and 0.8 µg/µL. The mice were randomly divided into 5 groups: (1) the NOR group; (2) the CRVO group (PBS, 1 µL/eye); (3) the 0.2 SP + CRVO group (0.2 µg/µL SP, 1 µL/eye); (4) the 0.4 SP + CRVO group (0.4 µg/µL SP, 1 µL/eye); and (5) the 0.8 SP + CRVO group (0.8 µg/µL SP, 1 µL/eye). The mice in the CRVO-related groups were anesthetized via an intraperitoneal injection of 1% sodium pentobarbital (50 mg/kg), after which tropicamide phenylephrine (China Santen Pharmaceutical Co., Ltd., Suzhou, China) was used for pupil dilation, and oxybuprocaine hydrochloride (China Santen Pharmaceutical Co., Ltd., Suzhou, China) was used for topical anesthesia in the right eyes. The corresponding solution was then intravitreally injected into the right eye of each mouse via a 33-gauge needle mounted on a Hamilton syringe (Sigma‒Aldrich, St. Louis, MO, USA). The needle remained in the vitreous cavity for 15 s and was gently withdrawn to avoid leakage of the injected solution. Ofloxacin drops were topically administered immediately and at 24 h after the injection to prevent infection. The NOR group did not receive any treatment.

### 2.3 Mouse model of laser-induced CRVO

Two days after the intravitreal injection, the mice in the CRVO-related groups were subjected to systemic anesthesia, mydriasis and topical anesthesia of the right eyes. Rose Bengal (5 mg/mL, dissolved in normal saline, filtered, and kept in the dark; Sigma‒Aldrich, St. Louis, MO, USA) was injected at 50 mg/kg through a tail vein. All retinal veins in the right eye were immediately photocoagulated via a 532 nm laser (irradiation duration: 1 s; irradiation interval: 200 ms) generated by a Micron III (Phoenix MICRON, Bend, OR, USA). In accordance with the literature (34, 35), the laser energy was set at 50 mW, 100 mW, and 150 mW, and the laser energy with the most intense photocoagulation effect on retinal veins was selected. A total of 10–12 photocoagulations were performed at 3 sites along the trajectory of each vein. The coagulation sites were at least 1.5-disc diameter away from the optic disc. The fundus image of each mouse was captured to confirm occlusion, and the morphological characteristics of the coagulated veins were recorded. The mice in the NOR group received no treatment.

### 2.4 Fluorescein fundus angiography

Fluorescein fundus angiography (FFA) was not performed on the day (day 0) when Rose Bengal injection and laser photocoagulation were conducted to avoid potential interactions between fluorescein and Rose Bengal. Therefore, FFA was performed 3, 6, 9, and 12 days after laser photocoagulation. Briefly, systemic and topical anesthesia and mydriasis were performed as described above. Levofloxacin gel was applied, 0.5% fluorescein (Sigma‒Aldrich, St. Louis, MO, USA; dissolved in sterile normal saline) was subcutaneously injected as previously described (36, 37), and FFA fundus images were captured with identical optical parameters using the fundus imaging module of the Micron III (Phoenix MICRON, Bend, OR, USA).

On day 3 post photocoagulation, the reperfused retinal veins were identified by comparing the morphology and trajectory of the retinal vessels on the FFAs with those on the fundus images on day 0. The reperfused retinal veins at other time points were determined by comparing the FFA images at a certain time point with earlier images. The reperfusion rate was calculated as the percentage of reperfused veins over total photocoagulated veins. On the other hand, customized software was developed to quantify the intravascular and extravascular intensities of sodium fluorescein in the FFA images. Briefly, the vascular contours in the FFA images were manually labeled, and machine learning algorithms were employed to develop software to automatically identify and label the blood vessels in the FFA images. Moreover, the software calculates intravascular fluorescence intensity values, reflecting the patency of retinal vessels. Additionally, the software calculates the value of extravascular fluorescence intensity, indicating the extent of retinal vessel leakage. The leakage area was also analyzed by ImageJ software (National Institute of Health, Bethesda, MD, USA) and is presented as the percentage of the leakage area to the total image area.

### 2.5 Optical coherence tomography

Optical coherence tomography (OCT, Heidelberg Engineering GmbH, Heidelberg, Germany) was performed on the mice 3, 6, 9, and 12 days after laser photocoagulation of the retinal veins. Systemic and topical anesthesia and mydriasis were performed as described above, and levofloxacin gel was applied. The OCT device requires an appropriate focal length to visualize the retina and the optic disc. The position of the instrument and parameter settings were adjusted to place the optic disc at the center of the area to be scanned, and the horizontal scan mode was selected to obtain 19 equidistant sectional images of this area, with each image being overlaid by 9 scans (HRT = 9). One of the 19 images was used as the representative OCT of each retina (Supplementary Figure 1). Retinal thickness was analyzed using built-in software in the OCT device (Supplementary Figure 2).

### 2.6 Electroretinogram

On the basis of the FFA and OCT results, only an intravitreal injection of recombinant SP at a high dose (0.8 μg/*μ*L) was associated with significantly aggravated retinal lesions, such as delayed reperfusion and exacerbated vascular leakage and retinal edema, in the CRVO mouse model. Therefore, electroretinogram (ERG) was performed to assess changes in retinal electrophysiological functions associated with intravitreal SP exposure in the NOR, CRVO, and 0.8 SP + CRVO groups of mice on the 9th day after laser photocoagulation, when the aggravation peaked. Briefly, the mice were dark-adapted overnight (14∼16 h) before ERG was performed. On the day of the experiment, the mice were anesthetized as described above. Ofloxacin gel was topically applied to avoid ocular surface desiccation. The mice were secured in a prone position on an examination stage, with a ground electrode placed close to the tail and a reference electrode to the forehead. Then, a platinum needle electrode was positioned in gentle contact with the cornea of the right eye. Following stabilization of the baseline voltage (± 10 mV), the full-field dark-adapted ERGs elicited by flash lights (white, 0.01, 3.0, and 30.0 cd.s/m^2^) were recorded with RetiMINIR Visual Electrophysiology (ChongQing IRC Medical Equipment Co., Ltd., Chongqing, China). The mice were then adapted for 30 min to a white background light of 30 cd.s/m^2^, and the full-field light-adapted ERGs induced by a single flash (white, 3.0 cd.s/m^2^) and a 30 Hz flicker (white, 3.0 cd.s/m^2^) under a white background light of 30 cd.s/m^2^ were recorded.

### 2.7 10× genomics scRNA sequencing

#### 2.7.1 Sample collection

Since vascular reperfusion, retinal edema, and electrophysiological functions in retinas of 0.8 SP + CRVO group of mice were significantly aggravated compared to those of CRVO group of mice on the 9th day post-photocoagulation, retina samples from these two groups were collected at this time point and analyzed by scRNA sequencing to explore the cellular and molecular clues associated with the aggravation of retinal lesions. In brief, the mice were euthanized by an intraperitoneal injection of sodium pentobarbital (100 mg/kg). The right eye of each mouse was carefully removed and rapidly placed in prechilled PBS. The muscles and fascia around the eyeball were removed, and the retina was gently isolated with sharp tweezers under a dissection microscope. Five isolated retinas from each group were pooled, washed with Dulbecco's phosphate-buffered saline and immediately placed in prechilled tissue preservation solution to maintain their biological activity. The retina samples were transported to Novogene Co., Ltd. (Beijing, China) via a cold chain for further processing and analysis.

#### 2.7.2 Single-cell suspensions

The retina samples were minced in cold PBS and thoroughly digested with gentle agitation at 37 °C for 30–90 min. Then, the digestion product was collected and processed via stringent cell filtration, extensive washing, low-speed centrifugation, and density gradient centrifugation following the manufacturer's protocols (10× Genomics, Pleasanton, CA, USA), after which the tissue debris, dead cells, and cell clumps were effectively removed. The final single-cell suspension contained more than 500,000 viable cells with diameters of 5–30 μm and a cell viability above 80%.

#### 2.7.3 Construction of the cDNA library and sequencing

A single-cell suspension at an appropriate concentration (700–1200 cells/μL) was mixed with reverse transcription reagents and then loaded onto the chromium microfluidic chip. The barcoded hydrogel beads and oil phase were added to the corresponding wells of the chip. After formation of a stable water-in-oil structure, reverse transcription was performed in a 10× Chromium Controller (10× Genomics, Pleasanton, CA, USA) at 42 °C for 90 min, after which the mixture was inactivated at 80 °C for 15 min. Then, a sequencing library was constructed using the Chromium Single Cell 3' Reagent Kits v2 (10× Genomics, Pleasanton, CA, USA), and sequencing was performed on a HiSeq PE150 platform (Illumina, San Diego, CA) according to the manufacturer's procedures.

### 2.8 Analysis of scRNA sequencing data

#### 2.8.1 Quality control of the raw data

The raw sequencing images were converted into reads in FASTQ format using CASAVA. Each read is 150 bp in length, and the first 26 bp contain a cell barcode and unique molecular identifier (UMI), which are the key information on cell origin and quality control, respectively. FastQC was used to perform quality control of the FASTQ reads, including removing reads with low-quality tails or excessive N bases, eliminating adapter sequences, and deleting reads less than 26 bp in length.

#### 2.8.2 Alignment with the reference genome and cell filtering

High-quality FASTQ reads were subsequently processed using Cell Ranger (10× Genomics, Pleasanton, CA, USA) for precise alignment to the reference genome. The aligned reads were annotated and quantified on the basis of the corresponding GTF gene annotation file, generating a cell-by-gene expression matrix that contained unique gene expression profiles for each cell. To further improve data quality, rigorous cell filtering, based on the number of detected genes and the proportion of mitochondrial UMIs, was performed on the expression matrix.

#### 2.8.3 Clustering analysis

The Seurat package was used to normalize the data and select highly variable genes across cells. Principal component analysis was then conducted on these highly variable genes with a clustering resolution of 0.6. The principal components with the greatest contribution to intercellular heterogeneity were selected, and the Louvain algorithm was used for clustering analysis. The clustering results were visualized via t-distributed stochastic neighbor embedding (t-SNE).

#### 2.8.4 Gene expression analysis

To characterize gene expression patterns among distinct cell clusters, the Seurat package was used to compare the gene expression profile of each specific cell cluster with those of other cell clusters and generate a list of highly expressed genes, which provided crucial evidence for subsequent cell cluster annotation.

#### 2.8.5 Annotation of cell clusters

The highly expressed genes of each cell cluster were compared with the marker genes recorded in well-established public databases, such as CellMarker, Mouse Cell Atlas, PanglaoDB, and CancerSEA, as well as with the cell type-specific marker genes reported in the literature. After assessing the expression abundance of highly expressed genes within a certain cell cluster and their consistency with documented marker genes, the cell cluster identity was determined.

#### 2.8.6 Analysis of differentially expressed genes in each cell cluster

Cell Ranger was used to quantify the gene expression profiles of the identified cell clusters. For each cell cluster, gene expression levels were compared among different experimental groups to screen for differentially expressed genes (DEGs). The Benjamini–Hochberg procedure was applied to correct for multiple testing in the scRNA sequencing data. The threshold criteria for DEG identification were defined as |log_2_(fold change)| ≥ 0.25 and adjusted *p* < 0.05.

#### 2.8.7 Enrichment analysis of DEGs in each cell cluster

The DEGs in each cell cluster were subjected to Gene Ontology (GO) functional enrichment analysis and Kyoto Encyclopedia of Genes and Genomes (KEGG) pathway enrichment analysis. Furthermore, the STRING database was used to construct protein–protein interaction (PPI) networks of the DEGs to explore their potential molecular interactions.

### 2.9 IF

#### 2.9.1 Staining of paraffin sections

On the 9th day following laser photocoagulation, the eyeballs of the mice were removed after the animals were euthanized with an overdose of sodium pentobarbital (100 mg/kg). The eyeballs were fixed in acidic fixative and embedded in paraffin. Five sections were prepared (5 μm/section) around the optic nerve of each eyeball. The sections were cleared, dewaxed, and dehydrated with gradient alcohol. Antigen retrieval was performed by boiling the sections in 0.01 mol/L sodium citrate buffer (pH 6.0; Beijing Zhongshan Golden Bridge Biotechnology Co., Ltd., Beijing, China) for 20 min. Then, the sections were washed with PBS and blocked with 10% goat serum (Beijing Zhongshan Golden Bridge Biotechnology Co., Ltd., Beijing, China) at room temperature (RT) for 2 h. The sections were incubated with a mouse monoclonal anti-CRALBP antibody (1:1000) and a rabbit polyclonal anti-LGSN antibody (1:1000) (Thermo Fisher Scientific, Waltham, MA, USA) at 4  °C overnight. The next day, the sections were extensively washed and incubated with Alexa 647-conjugated goat anti-mouse and Alexa 488-conjugated goat anti-rabbit secondary antibodies (both 1:1,000) at RT for 1 h. After extensive washing, the coverslips were mounted on the sections with Prolong anti-Fade with 4',6-diamidino-2-phenylindole (DAPI) (Vector Laboratories, Burlingame, CA, USA). The sections were kept at RT in the dark overnight. Images were captured under a Zeiss LSM800 confocal microscope (Carl Zeiss AG, Oberkochen, Germany) with cellSens Standard software (Olympus Optical, Tokyo, Japan) using identical optical parameters.

#### 2.9.2 Staining of retinal whole mounts

On the 9th day after laser photocoagulation, the right eyes were removed from the groups of mice after euthanization. The eyes were fixed in 4% paraformaldehyde (PFA) for 40 min, and the retinas were isolated as described above. The isolated retinas were fixed in 4% PFA at 4 °C overnight, washed with PBS, and permeabilized with permeabilization buffer (pH 6.8, 1% BSA, 0.5% Triton X-100 in PBS) at 4 °C overnight. After washing with PBlec buffer (pH 6.8, 1% Triton X-100, 0.1 mM CaCl_2_, 0.1 mM MgCl_2_, 0.1 mM MnCl_2_ in PBS), the retinas were stained with the primary antibodies and the corresponding secondary antibodies as described above. Each retina was cut into 3∼4 petals and mounted on slides, with the photoreceptor side facing down, using Prolong anti-Fade with DAPI (Vector Laboratories, Burlingame, CA, USA). The slides were incubated at RT in the dark overnight prior to observation under a confocal fluorescence microscope (Zeiss, Oberkochen, Germany). One image was captured on each retina petal by cellSens Standard software (Olympus Optical, Tokyo, Japan) using identical optical parameters. The number of cells that were positive for either CRALBP or LGSN or both was recorded using Adobe Photoshop CS5 (Adobe Systems, San Jose, CA, USA). The number of CRALBP^+^ cells per image and the percentage of CRALBP^+^/LGSN^+^ cells over total CRALBP^+^ cells were calculated. The MFI of the LGSN signal in total CRALBP^+^ cells was quantified using ImageJ software (National Institute of Health, Bethesda, MD, USA).

### 2.10 Cell culture and treatments

A human spontaneously immortalized retinal Müller cell line (MIO-M1) was purchased from the cell bank of Blue Flag Biotechnology (Shanghai, China) and maintained in Dulbecco's modified Eagle's medium (DMEM, Cat No. C11965500BT; Thermo Fisher Scientific, Waltham, MA, USA) supplemented with 10% fetal bovine serum (Thermo Fisher Scientific, Waltham, MA, USA), 100 U/mL penicillin, 100 µg/mL streptomycin (Thermo Fisher Scientific, Waltham, MA, USA), and 2 mM L-glutamine (Thermo Fisher Scientific, Waltham, MA, USA) at 37 °C and 5% CO_2_ in a humidified incubator. Cells at passage 3 were used for the experiments. Briefly, the cells were seeded in 12-well plates at a density of 4 × 10^5^ cells/well. Recombinant SARS-CoV-2 SP at concentrations ranging from 0.01 μg/mL to 1 μg/mL has been shown to effectively induce gene expression changes in neuroglial cells (38–40). Hence, the cells were randomly divided into 4 groups: (1) the serum-free (SF) group: the cells were incubated with DMEM without glutamine (Cat No. 11960-044; Thermo Fisher Scientific, Waltham, MA, USA); (2) the SF + 0.01 SP group: the cells were incubated with glutamine (-) DMEM containing 0.01 μg/mL SP; (3) the SF + 0.1 SP group: the cells were incubated with glutamine (-) DMEM containing 0.1 μg/mL SP; (4) the SF + 1 SP group: the cells were incubated with glutamine (-) DMEM containing 1 μg/mL SP. The SF condition was used to mimic the microenvironment in the retina under CRVO. After 12 h, the cells were harvested for gene expression analysis.

### 2.11 RNA extraction and reverse transcription

Total RNA was extracted using an RNA Universal Animal Kit (Tiangen, Beijing, China) according to the manufacturer's protocol. The concentration and purity of the total RNA were determined by a Nanodrop 2,000 (Thermo Fisher Scientific, Waltham, MA, USA). After digestion with DNase I at 37 °C for 30 min, the total RNA was reverse transcribed by a RevertAid First Strand cDNA Synthesis Kit using random hexamer primers (Thermo Fisher Scientific, Waltham, MA, USA).

### 2.12 Quantitative real-time PCR

Quantitative real-time PCR (qPCR) was performed on a Roche 4,800 (Roche, Basel, Switzerland). The mouse glyceraldehyde 3-phosphate dehydrogenase (*Gapdh*) gene served as an internal standard. The 25-μL reaction mixture was composed of 4 μL of cDNA template, gene-specific qPCR primers (Table 1), and 12.5 μL of EvaGreen 2× Master Mix (ABM, Richmond, BC, Canada). The qPCR program consisted of preincubation at 50 °C for 2 min, denaturation at 95 °C for 10 min, 40 cycles of denaturation at 95 °C for 15 s and extension at 60 °C for 1 min. A dissociation stage was added to check amplicon specificity. The relative expression levels of the genes of interest were analyzed using a comparative threshold cycle (2^−*ΔΔ*Ct^) method. The qPCR results revealed dose-dependent upregulation of *LGSN* gene expression in human retinal Müller cells stimulated with different concentrations of SP, with 1 μg/mL SP being the most effective. Therefore, the following experiments were performed in Müller cells under stimulation with 1 μg/mL SP.

**Table 1:** Primers used in this study.

Gene symbol	Species	Accession number	Sequences
*LGSN*	human	NM_016571.3	FP: GCCTCCAGTTTGTACGATTTGAA
			RP: TGAAAAAAGTGTGCAGGGATAGTC
*IL1B*	human	M15330.1	FP: CACGATGCACCTGTACGATCA
			RP: AGACATCACCAAGCTTTTTTGCT
*IL6*	human	M54894.1	FP: GCTGCAGGCACAGAACCA
			RP: ACTCCTTAAAGCTGCGCAGAA
*TNF*	human	NM_000594.4	FP: TGGCCCAGGCAGTCAGA
			RP: GGTTTGCTACAACATGGGCTACA

### 2.13 Measurement of intracellular glutamine levels

MIO-M1 cells were seeded in 6-well plates at a density of 2 × 10^6^ cells/well. The cells were randomly divided into 2 groups: (1) the SF group, in which the cells were incubated with glutamine (-) DMEM; and (2) the SF + 1 SP group, in which the cells were incubated with glutamine (-) DMEM containing 1 μg/mL SP. Twelve hours after treatment, the cells were collected, and total protein was extracted with a tissue protein extraction kit (CWBIO, Beijing, China).

The concentration of total protein was determined using a Bicinchoninic Acid (BCA) Protein Assay Kit (CWBIO, Beijing, China). A standard of 2 mg/mL bovine serum albumin (CWBIO, Beijing, China) was serially diluted to generate a standard curve and served as a positive control for protein quantification. The diluent was included as a negative control. Afterwards, 25 μL of the standards and the 7-fold diluted protein samples were incubated with the BCA reagents at 37 °C for 30 min. The absorbance at 562 nm was measured. The protein concentration (μg/μL) of each sample was determined from the standard curve and multiplied by the dilution factor.

Each extracted total protein sample was applied to an Amicon Ultra-0.5 Centrifugal Filter Unit (50 kDa MWCO, Sigma‒Aldrich, St. Louis, MO, USA) and centrifuged at 5,000 g and 4 °C for 60 min. The filtrates were enriched with molecules lower than 50 kDa. The glutamine levels in the filtrates were measured using a Glutamine (Gln) Colorimetric Assay Kit (Elabscience Biotechnology, Wuhan, China) according to the manufacturer's instructions. The intracellular glutamine concentration (μM) was normalized to the total protein concentration (μg/μL).

### 2.14 Cell viability assay

MIO-M1 cells were seeded in 96-well plates at a density of 8 × 10^3^ cells/well. The cells were randomly divided into the SF group and SF + 1 SP group. A cell viability assay was performed 12 h after SP stimulation via a Cell Counting Kit-8 (Dojindo Laboratories, Kumamoto, Japan). The cells were incubated with glutamine (-) DMEM containing 10% CCK-8 solution for 1 h at 37 °C. The absorbance at 450 nm was measured using an Infinite 200 PRO Multimode Microplate Reader (Tecan Group Ltd., Männedorf, Switzerland) and is presented as a percentage (%) of normal controls.

### 2.15 Statistics

The data were statistically analyzed via GraphPad Prism 10.1.2 software (GraphPad Software, San Diego, CA, USA). The data were examined by D'Agostino and Pearson omnibus normality tests to confirm a Gaussian distribution, after which they were examined by Levene's test to confirm homogeneity of variance. All the data are expressed as the means ± SEMs. Differences between 2 experimental groups were analyzed by an unpaired t test; differences between multiple groups were assessed by one-way ANOVA or repeated-measures ANOVA as appropriate, with pairwise comparisons performed using Tukey's *post hoc* test. Statistical significance was set at *p* < 0.05.

## Results

### 3.1 A mouse model of CRVO was established by laser photocoagulation

**Figure 1:**
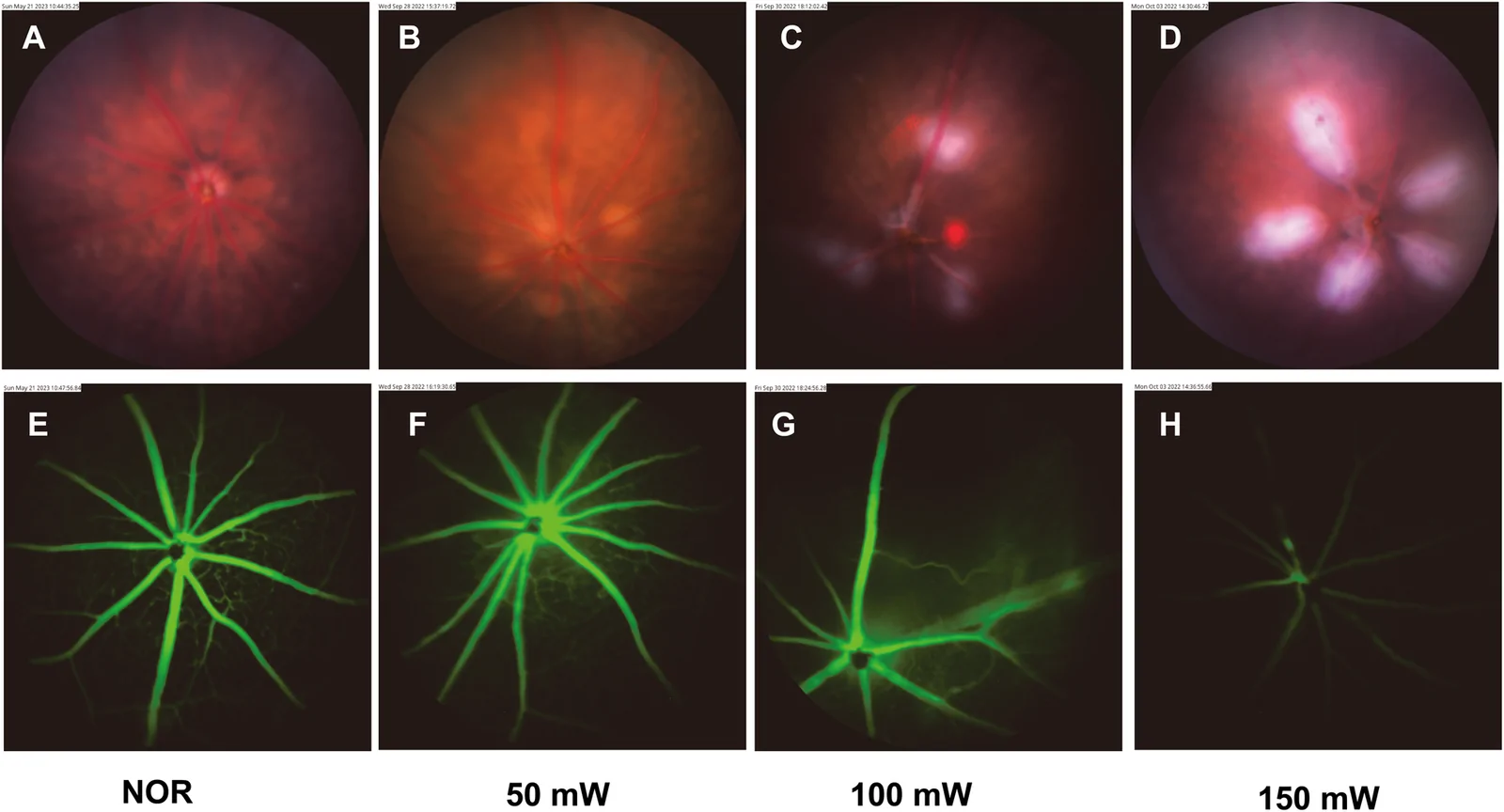
Establishment of a laser-induced mouse model of CRVO. Representative fundus images of mice receiving no treatment **(A)** or photocoagulation at all retinal veins with laser energy set at 50 mW **(B)**, 100 mW **(C)**, and 150 mW **(D)** Representative FFAs of the mice receiving no treatment **(E)** or photocoagulation at all retinal veins with laser energy set at 50 mW **(F)**, 100 mW **(G)**, and 150 mW **(H)**.

The energy of the 532 nm laser was set at 50, 100, and 150 mW with an irradiation duration of 1 s and an irradiation interval of 200 ms. The fundus image demonstrated that as the laser energy increased, the laser spots became increasingly obvious, and the retinal veins became whitish (Figures 1A–D); embolic points were observed. In parallel, FFA revealed an energy-dependent reduction in fluorescein dye filling in retinal vessels (Figures 1E–H); in particular, treatment with a laser with a 532-nm wavelength and 150-mW energy caused almost no filling of the fluorescein dye in retinal vessels (Figure 1H), indicating nearly complete blockage of blood circulation in the retina. Therefore, a 150-mW laser at 532 nm with a 1 s irradiation duration and a 200 ms irradiation interval was selected to establish a mouse model of CRVO in this study.

### 3.2 CRVO mice pretreated with high-dose SP delayed reperfusion of occluded retinal veins

**Figure 2:**
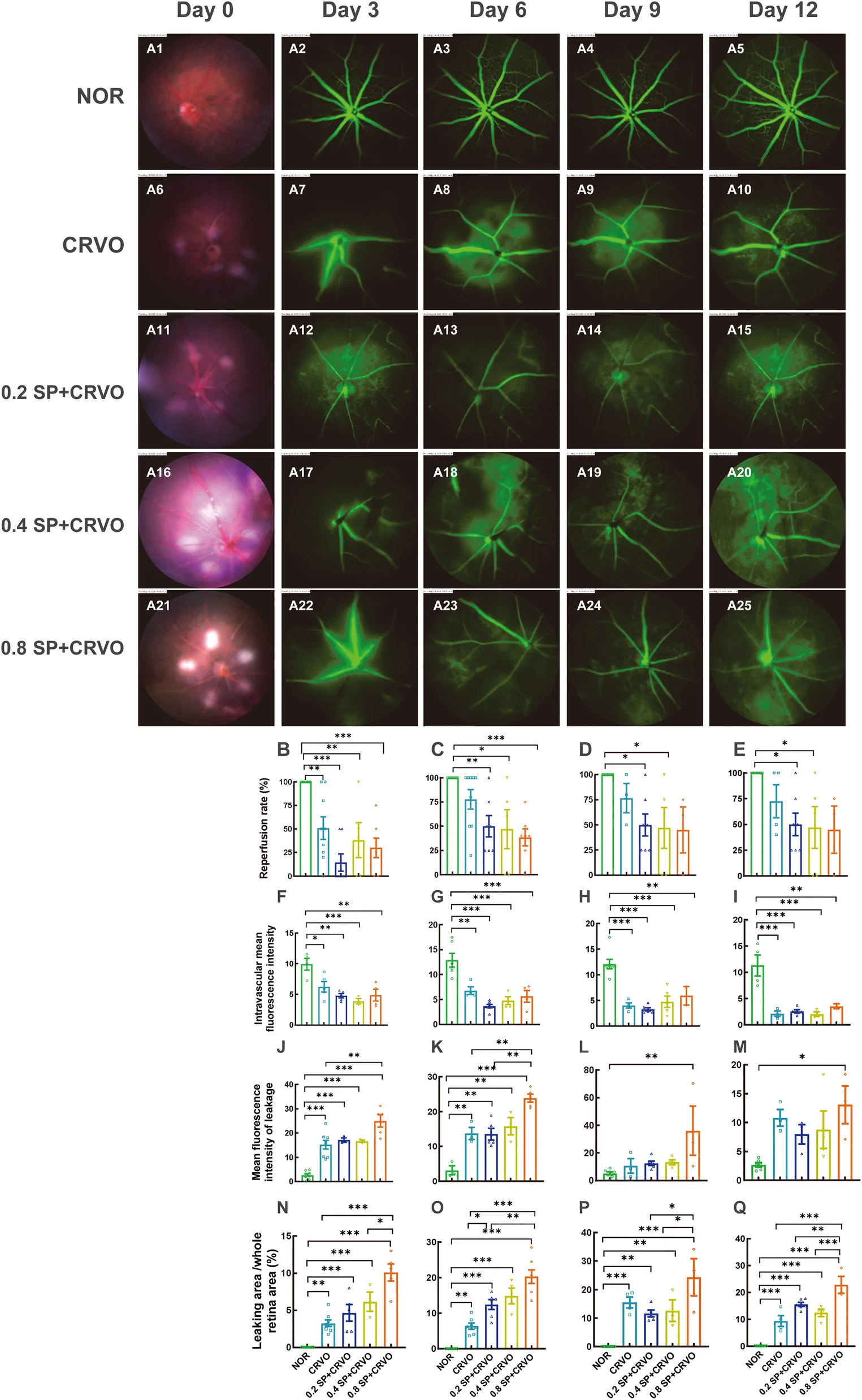
Effects of intravitreal injection of SP at various concentrations on the retinal vasculature in the CRVO model. Representative fundus images and FFAs from each group of mice at 0, 3, 6, 9, and 12 days after laser photocoagulation are shown in (A1–A25). The reperfusion rates of retinal vessels at 3 **(B)**, 6 **(C)**, 9 **(D)**, and 12 **(E)** days after laser photocoagulation were quantified. The intravascular MFIs at the corresponding time points after laser photocoagulation were quantified **(F–I)**. The MFIs of leakage **(J–M)** and the percentages of the leakage area over the total retinal area **(*N*–Q)** at different time points after photocoagulation are also shown. *n* = 3–9. The data are presented as the means ± SEMs. *p < 0.05, **p < 0.01, ***p < 0.001; one-way ANOVA followed by Tukey's *post hoc* test.

The number of reperfused retinal vessels on day 3 following CRVO induction was determined by comparing the fundus images on day 0 (Figures 2A1,A6,A11,A16,A21) with the FFA images on day 3 (Figures 2A2,A7,A12,A17,A22). On day 3 after laser photocoagulation, the reperfusion rate of the CRVO group was 50.89 ± 12.66%, which was significantly lower than that of the normal control group (*p* < 0.01, NOR vs. CRVO; Figures 2A2, A7, 2B). The reperfusion of the retinal veins was delayed by preinjection of SP at different concentrations, with reperfusion rates of 14.29 ± 13.58%, 38.00 ± 15.09%, and 30.00 ± 13.58% in the 0.2 SP + CRVO group, 0.4 SP + CRVO group, and 0.8 SP + CRVO group, respectively (Figures 2A12,A17,A22,2B). However, no significant difference in the reperfusion rate was detected between the SP-injected and noninjected CRVO groups (Figure 2B; all *p* > 0.05). On day 6 after laser-induced coagulation, the reperfusion rate in the CRVO group reached 77.78% ± 12.66%, whereas the reperfusion rates in the 0.2 SP + CRVO group, 0.4 SP + CRVO group, and 0.8 SP + CRVO group were 50.00% ± 13.58%, 47.00% ± 15.09%, and 38.57% ± 13.58%, respectively (Figures 2A8,A13,A18,A23,C). Notably, the reperfusion rate in the 0.8 SP + CRVO group was significantly lower than that in the CRVO group (Figure 2C; 0.8 SP + CRVO vs. CRVO, *p* < 0.05). In each experimental group, the retinal vessel reperfusion rates remained essentially unchanged on days 9 and 12 post photocoagulation compared with those on day 6 (Figures 2A4,A9,A14,A19,A24,A5,A10,A15,A20,A25,C–E; all *p* > 0.05). Therefore, in the laser-induced CRVO mouse model, the retinal veins started reperfusion on day 3 after photocoagulation, and the reperfusion rate increased over time until day 9 after coagulation. Compared with the CRVO group, the 0.8 SP + CRVO group exhibited more severe vessel occlusion and more delayed reperfusion.

### 3.3 CRVO mice pretreated with high-dose SP did not further reduce intravascular filling of fluorescein sodium

After laser treatment, the blood flow in the photocoagulated vessels was obstructed; hence, the amount of fluorescein sodium in those vessels was dramatically reduced. Compared with the NOR group, the CRVO group presented a time-dependent reduction in the intravascular mean fluorescence intensity (MFI), with the intravascular MFI on day 12 following the laser treatment being merely 19.70% of that in the normal controls (Figures 2A2–A5,2A7–A10,F–I). The intravascular MFI of the CRVO groups with SP prestimulation also showed similar trends over time (Figures 2A12–A15,2A17–A20,2A22–A25,2F–I). However, there was no significant difference in the intravascular MFI between the CRVO groups with SP pretreatment and the CRVO group without pretreatment at any time point (Figures 2A7–A10,2A12–A15,2A17–A20,2A22–A25,F–I; all *p* > 0.05).

### 3.4 CRVO mice pretreated with high-dose SP exhibited exacerbated vascular leakage in retinas

On days 3 and 6 after laser-induced photocoagulation, the MFIs of retinal vessel leakage in the CRVO group and the SP + CRVO group were significantly greater than those in the NOR group (all *p* < 0.001 on day 3 post coagulation; *p* < 0.01 for CRVO, 0.2 and 0.4 SP + CRVO vs. NOR; *p* < 0.001 for 0.8 SP + CRVO vs. NOR on day 6 post coagulation; Figures 2A2,A7,A12,A17,A22,2A3,A8,A13,A18,A23,J–K). Moreover, the MFIs of leakage in the 0.8 SP + CRVO group were significantly greater than those in the NOR group at all time points (for 0.8 SP + CRVO vs. NOR, both *p* < 0.001 on day 3 and day 6 post coagulation; *p* < 0.01 on day 9 post coagulation; *p* < 0.05 on day 12 post coagulation; Figures 2A2–A5,2A22–A25; J–M) and were also significantly greater than those in the CRVO group on both day 3 and day 6 following photocoagulation (0.8 SP + CRVO vs. CRVO, both *p* < 0.01; Figures 2A7–A8,2A22–A23,J–K). However, there was no significant difference in the extravascular leakage MFI between the 0.2 or 0.4 SP + CRVO group and the CRVO group (Figures 2J–M).

The percentage of the fluorescence leakage area over the total retina area was employed as another proxy for evaluating vessel leakage in the CRVO mouse retina. The proportion of the leakage area in the CRVO group gradually increased and peaked on the 9th day after photocoagulation (Figures 2A7–A10,N–Q). On day 3 and day 6 post photocoagulation, the percentages of the fluorescence leakage area in the CRVO groups preinjected with different concentrations of SP were significantly greater than those in the normal controls and increased in a dose-dependent manner, culminating in the 0.8 SP + CRVO group (all *p* < 0.01, for the CRVO or SP + CRVO groups vs. NOR, Figures 2A2,A7,A12,A17,A22,2A3,A8,A13,A23,N–O). Notably, the fluorescence leakage area was significantly greater in the 0.8 SP + CRVO group than in the CRVO group throughout the experiment (all *p* < 0.001, 0.8 SP + CRVO vs. CRVO on days 3, 6, 9, and 12 post photocoagulation; Figures 2A7–A10,2A22–A25,N–Q).

### 3.5 CRVO mice pretreated with high-dose SP exhibited worsened retinal edema

**Figure 3:**
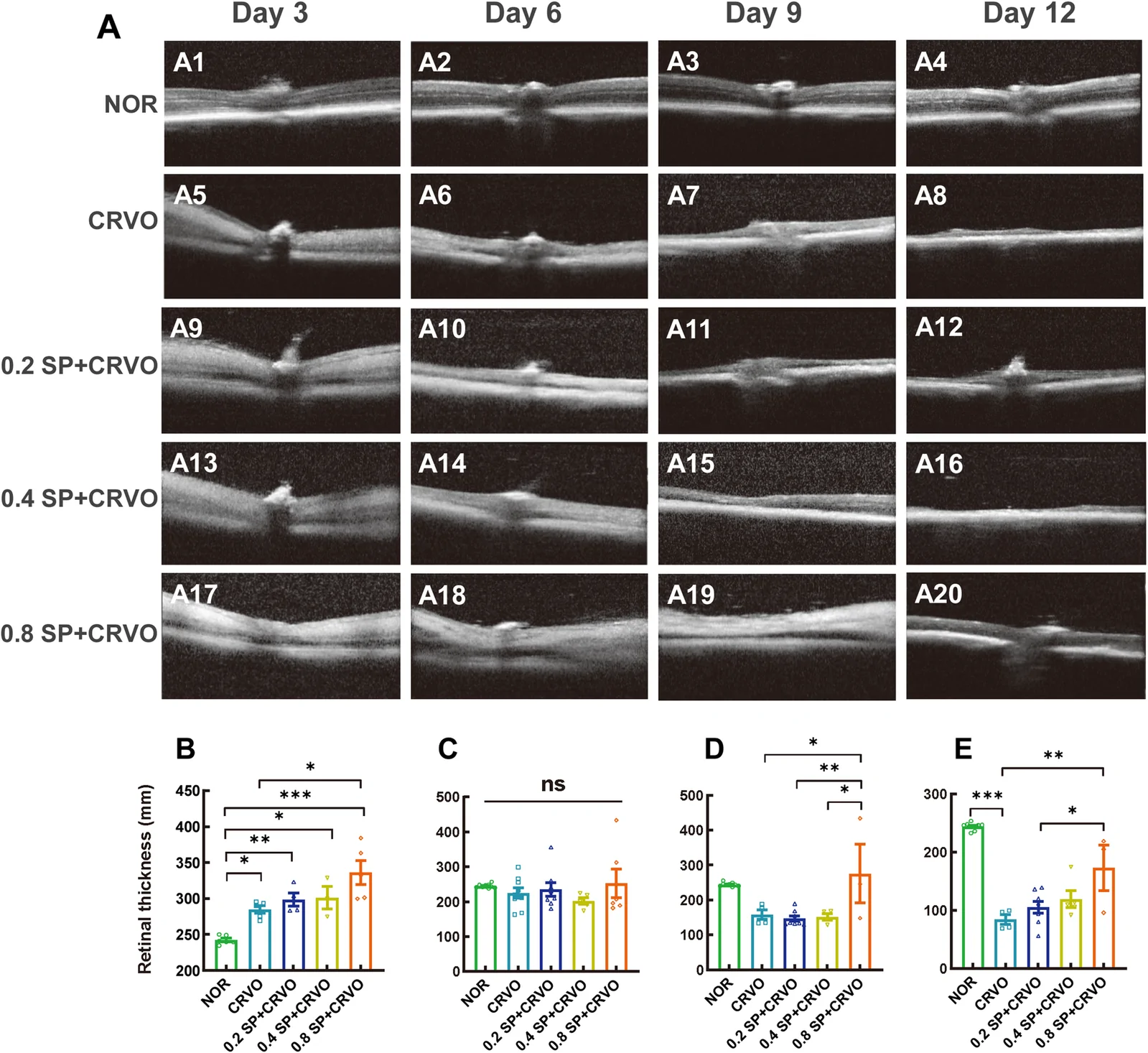
Effects of intravitreal injection of SP at different concentrations on retinal edema in the CRVO model. (A1–A20) Representative OCT images of each group at 3, 6, 9, and 12 days after laser photocoagulation. Retinal thickness was quantified at 3 **(B)**, 6 **(C)**, 9 **(D)**, and 12 **(E)** days after laser photocoagulation. *n* = 3–9. The data are presented as the means ± SEMs. *p < 0.05, **p < 0.01, ***p < 0.001; one-way ANOVA followed by Tukey's *post hoc* test.

Following laser-induced CRVO, OCT was employed to monitor retinal morphology. On day 3 post photocoagulation, the retinas of the NOR group exhibited intact structure and normal morphology, with a clear distinction of the retinal layers (Figures 3A1). In contrast, the retinas in the CRVO groups treated with or without SP displayed blurred layers and fluid accumulation in the subretinal spaces and neuroepithelial layers (Figures 3A5,A9,A13,A17). Moreover, the results revealed that the retinal thickness in the 0.8 SP + CRVO group was 1.18-fold greater than that in the CRVO group, and the thicknesses of both groups were significantly greater than those of the normal controls (*p* < 0.05, for CRVO vs. NOR; *p* < 0.001, for 0.8 SP + CRVO vs. NOR; Figure 3B). These results indicate the presence of retinal edema during the early phase following laser-induced CRVO, which could be exacerbated in the CRVO mice pretreated with high-dose SP.

Six days after photocoagulation, the retinal thickness in the CRVO groups with or without SP pretreatment began to decrease, resulting in no significant difference in retinal thickness among the experimental groups (all *p* > 0.05; Figures 3A2,A6,A10,A14,A18,C). However, blurred retinal layers and subretinal fluid were still visible in the CRVO-related retinas at this time point (Figures 3A6,A10,A14,A18). Nine days after photocoagulation, the retinal thicknesses in the CRVO group, 0.2 SP + CRVO group, and 0.4 SP + CRVO group continued to decrease; they decreased to 34.52%, 43.25%, and 48.91% of those in the normal controls, respectively, on day 12 post photocoagulation (Figures 3A3–A4,A7–A8,A11–A12,A15–A16,D–E). These observations suggest subsidence and even dissipation of retinal edema during the mid- and late phases of laser-induced CRVO; however, they may also indicate degeneration and tissue loss in the photocoagulated retinas that were pretreated with SP at low and middle dosages. In contrast, the 0.8 SP + CRVO retinas had the greatest thickness among the experimental groups from day 9 to day 12 following photocoagulation (both *p* < 0.05, for 0.8 SP + CRVO vs. CRVO, 0.8 SP + CRVO vs. 0.4 SP + CRVO; *p* < 0.01, for 0.8 SP + CRVO vs. 0.2 SP + CRVO; Figures 3A3–A4,A7–A8,A11–A12,A15–A16,A19–A20,D–E).

### 3.6 CRVO mice pretreated with high-dose SP demonstrated exacerbated retinal electrophysiological functions

**Figure 4:**
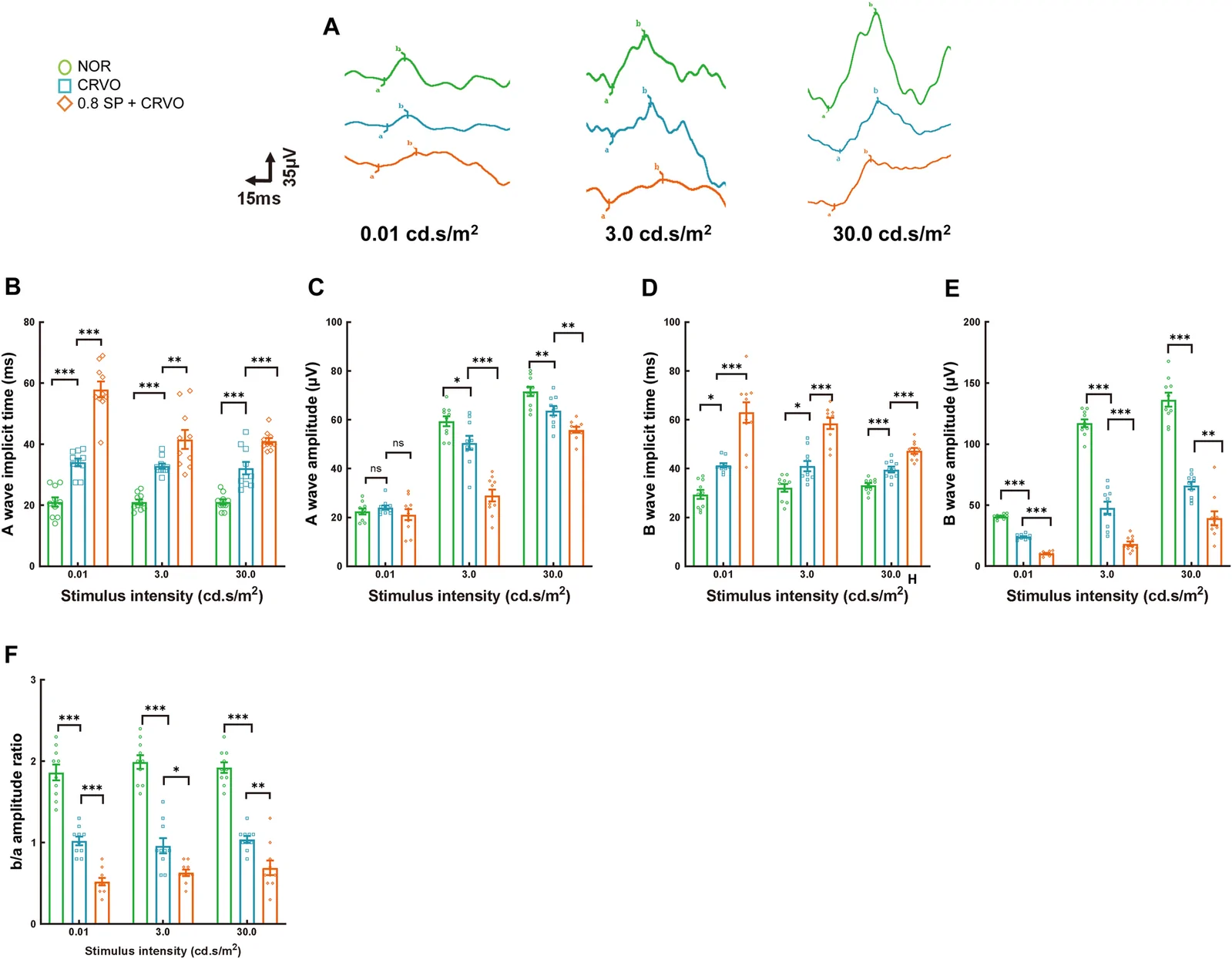
Effects of intravitreal injection of 0.8 μg SP on dark-adapted ERG in the CRVO model. **(A)** Representative traces of dark-adapted ERGs measured under light intensities of 0.01, 3.0, and 30.0 cd.s/m^2^ on the 9th day after laser photocoagulation in CRVO mice intravitreally pretreated with or without SP. **(B,C)** Quantification of a wave implicit time and amplitude of the dark-adapted ERGs under all light stimuli. **(D,E)** Quantification of b wave implicit time and amplitude of the dark-adapted ERGs. **(F)** Ratio of b wave amplitude over a wave amplitude (b/a) of the dark-adapted ERGs. *n* = 10. The data are presented as the means ± SEMs. *p < 0.05, **p < 0.01, ***p < 0.001; one-way ANOVA followed by Tukey's *post hoc* test.

Dark-adapted ERG was performed on the 9th day after photocoagulation in the NOR, CRVO, and 0.8 SP + CRVO groups of mice. The implicit time of both the a wave and b wave in CRVO mice was significantly longer than that of the normal controls under all light stimulus intensities (Figures 4A–D, all *p* < 0.05, NOR vs. CRVO), and the implicit time extension was exacerbated by intravitreal administration of 0.8 μg SP (Figures 4A–D, all *p* < 0.01, CRVO vs. 0.8 SP + CRVO). Moreover, the amplitudes of both waves in CRVO mice were significantly reduced compared to those in the normal controls (Figures 4A–E, all *p* < 0.05, NOR vs. CRVO) and were further diminished in the CRVO mice pretreated with SP (Figures 4A–E, all *p* < 0.01, CRVO vs. 0.8 SP + CRVO), except the a wave amplitude under the light intensity of 0.01 cd.s/m^2^, which showed no significant difference among experimental groups (Figure 4C, all *p* > 0.05). Consistently, the ratio of b wave amplitude over a wave amplitude (b/a) followed a similar trend to those of wave amplitudes under all light intensities (Figure 4F, all *p* < 0.05, NOR vs. CRVO, CRVO vs. 0.8 SP + CRVO), indicating the graded severity of retinal ischemia across these 3 experimental groups.

**Figure 5:**
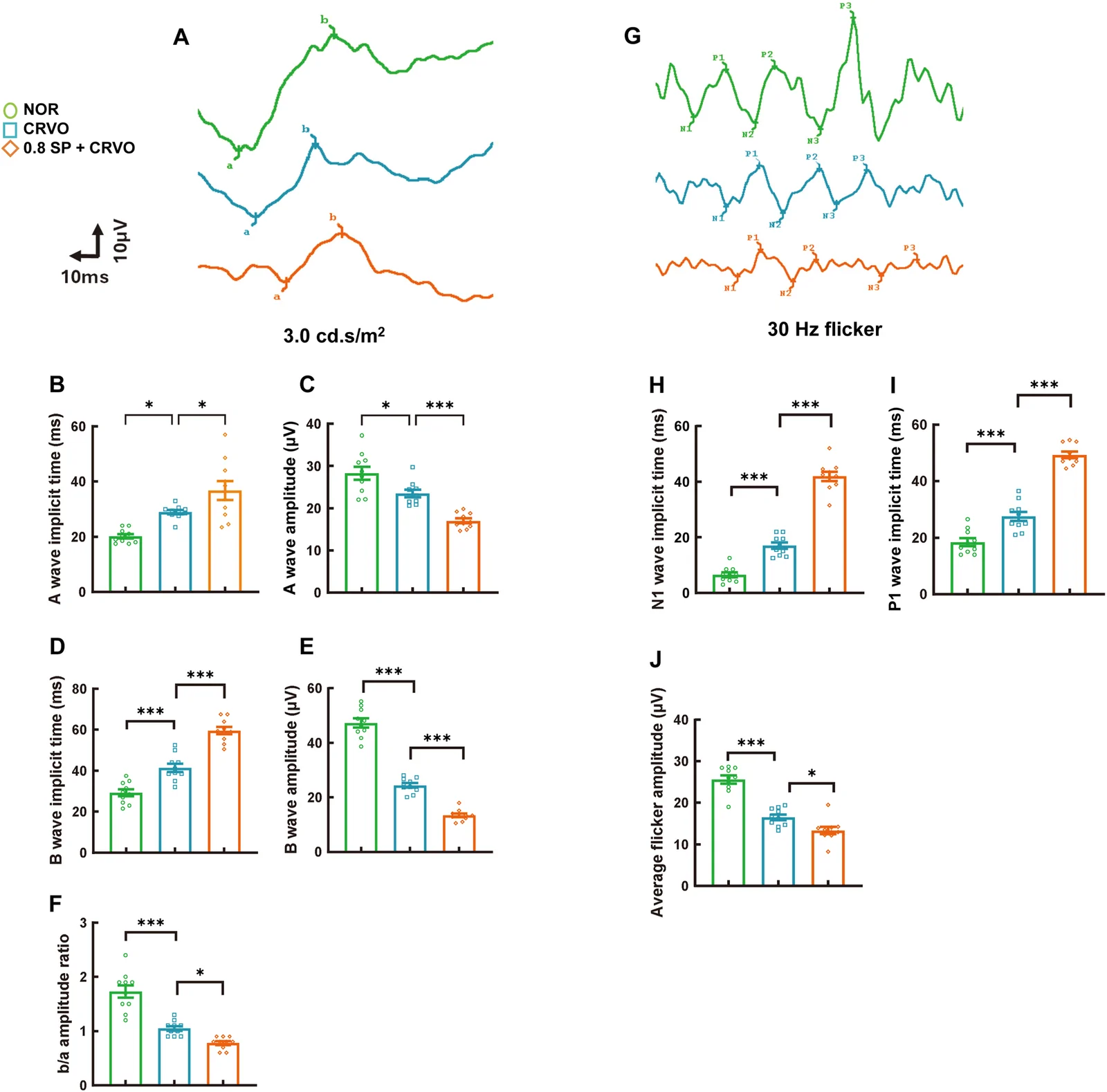
Effects of intravitreal injection of 0.8 μg SP on light-adapted ERG in the CRVO model. **(A)** Representative traces of light-adapted ERGs measured under 3.0 cd.s/m^2^ light intensity on the 9th day post-photocoagulation in CRVO mice pretreated with or without SP. **(B,C)** Quantification of a wave implicit time and amplitude of the light-adapted ERGs under 3.0 cd.s/m^2^ light stimulation. **(D,E)** Quantification of b wave implicit time and amplitude of the light-adapted ERGs under a 3.0 cd.s/m^2^ light stimulus. **(F)** b/a of the light-adapted ERGs under a 3.0 cd.s/m^2^ light stimulus. **(G)** Representative traces of the light-adapted ERGs under stimulation with a 30 Hz flicker. **(H,I)** Quantification of N1 and P1 wave implicit time in the light-adapted ERGs stimulated with the flicker. **(J)** Quantification of the average flicker amplitudes. *n* = 10. The data are presented as the means ± SEMs. *p < 0.05, ***p < 0.001; one-way ANOVA followed by Tukey's *post hoc* test.

In light-adapted ERG, a light stimulus of 3.0 cd.s/m^2^ induced similar changes, such as increasingly prolonged implicit time, decreased amplitudes, and lowered b/a ratio, among the NOR, CRVO, and 0.8 SP + CRVO groups (Figures 5A–F, all *p* < 0.05). Moreover, under the treatment of a 30 Hz flicker, the N1 wave implicit time in the CRVO retinas was prolonged 2.60-fold that in the normal retinas, and it was extended another 2.47-fold in the SP-pretreated CRVO retinas (Figures 5G,H, all *p* < 0.001, NOR vs. CRVO, CRVO vs. 0.8 SP + CRVO). Similar alterations were observed at the P1 wave implicit time (Figures 5G,I, all *p* < 0.001, NOR vs. CRVO, CRVO vs. 0.8 SP + CRVO). The average flicker amplitude was significantly reduced in CRVO mice (Figure 5J, *p* < 0.001, NOR vs. CRVO) and was further decreased by SP pretreatment (Figure 5J, *p* < 0.05, CRVO vs. 0.8 SP + CRVO). Taken together, these results suggest that when the retinal vascular lesions associated with intravitreal SP exposure reached the peak in the mouse model of CRVO, the retinal electrophysiological functions were also severely impaired.

### 3.7 ScRNA sequencing of the CRVO retinas

#### 3.7.1 Cell clusters in the retinas of the CRVO group and 0.8 SP + CRVO group

**Figure 6:**
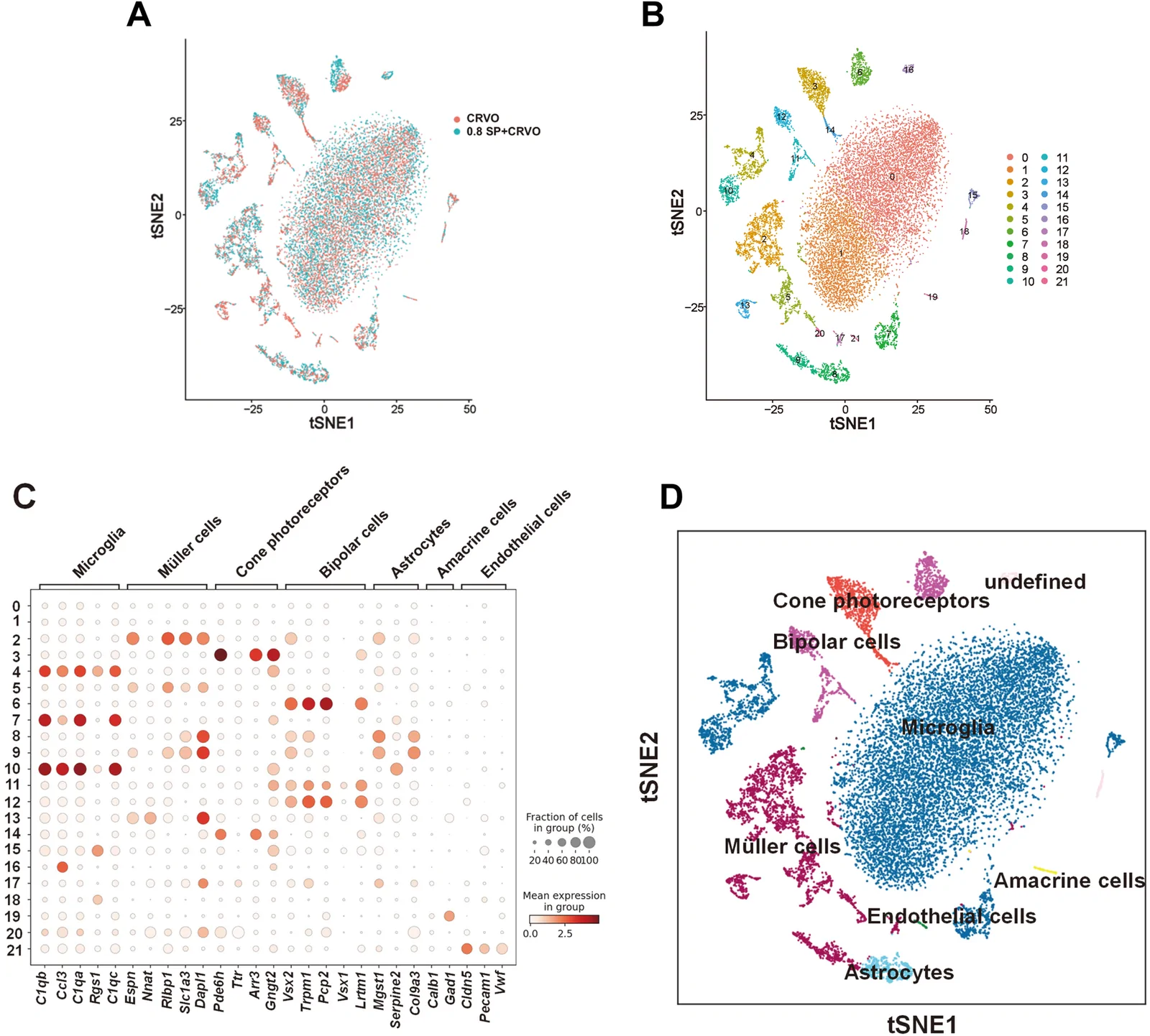
ScRNA sequencing of retinas from the CRVO group and 0.8 SP + CRVO group. **(A)** t-SNE plot of the CRVO and 0.8 SP + CRVO groups of retinas at a resolution of 0.6. **(B)** t-SNE plot of 22 identified cell clusters following data integration. **(C)** The average expression levels of marker genes in the 22 identified cell clusters and 7 annotated retinal cell types. **(D)** The distribution of the 7 annotated cell types in the CRVO retinas.

To explore the molecular and cellular clues regarding how intravitreal exposure to SP is associated with aggravated CRVO pathologies, retinas from the CRVO and 0.8 SP + CRVO groups were collected on the 9th day following photocoagulation and analyzed by scRNA sequencing. After quality control of the raw data was performed, 18,336 qualified cells were obtained. The data from the 2 groups were integrated, and a total of 22 cell clusters were identified (Figures 6A,B). Seven retinal cell types were annotated on the basis of the classical retinal marker genes listed in the databases and reported in the literature (41, 42), with each cell type containing one or more cell clusters (Figure 6C, Table 2). The distributions of the identified cell types among the cell clusters are shown in Figure 4D.

**Table 2:** Identification of cell types.

Retinal cell type	Marker genes	Cell clusters
Microglia	*C1qb, Ccl3, C1qa, Rgs1, C1qc*	0, 1, 4, 7, 10, 15
Müller cells	*Espn, Nnat, Rlbp1, Slc1a3, Dapl1*	2, 5, 9, 13, 17, 20
Cone photoreceptors	*Pde6h, Ttr, Arr3, Gngt2*	3, 14
Bipolar cells	*Vsx2, Trpm1, Pcp2, Vsx1, Lrtm1*	6, 11, 12
Astrocytes	*Mgst1, Serpine2, Col9a3*	8
Amacrine cells	*Calb1, Gad1*	19
Endothelial cells	*Cldn5, Pecam1, Vwf*	21

Cluster 16 was one of the undefined cell clusters. Compared with those in other cell clusters, the top 4 highly expressed genes in this cluster were *S100a9*, *S100a8*, *Cxcl2*, and *Srgn* (Supplementary Figure 3A). GO analysis of the highly expressed genes in cluster 16 revealed enrichment of these genes in the inflammatory response, cytokine production, adhesion plaque, actin adhesion, and cell adhesion molecule binding pathways (Supplementary Figure 3B). Similarly, the top 4 highly expressed genes in the other undefined cell cluster, cluster 18, included *Ccl5*, *Gzma*, *AW112010*, and *Ptprc* (Supplementary Figure 3C). Moreover, the results of the GO analysis revealed that the highly expressed genes in cluster 18 were enriched in cell functions related to T cell activation, cytoplasmic translation, cytoplasmic ribosomes, ribosome subunits, and the structure and molecular components of ribosomes (Supplementary Figure 3D).

Rod photoreceptors, retinal pigment epithelial cells, and retinal ganglion cells were not identified in the scRNA sequencing of CRVO and 0.8 SP + CRVO retinas. This might be due to severe damage to the retinal structure in the laser-induced CRVO model.

#### 3.7.2 Müller cells were substantially reduced in high-dose SP-pretreated CRVO retinas

**Figure 7:**
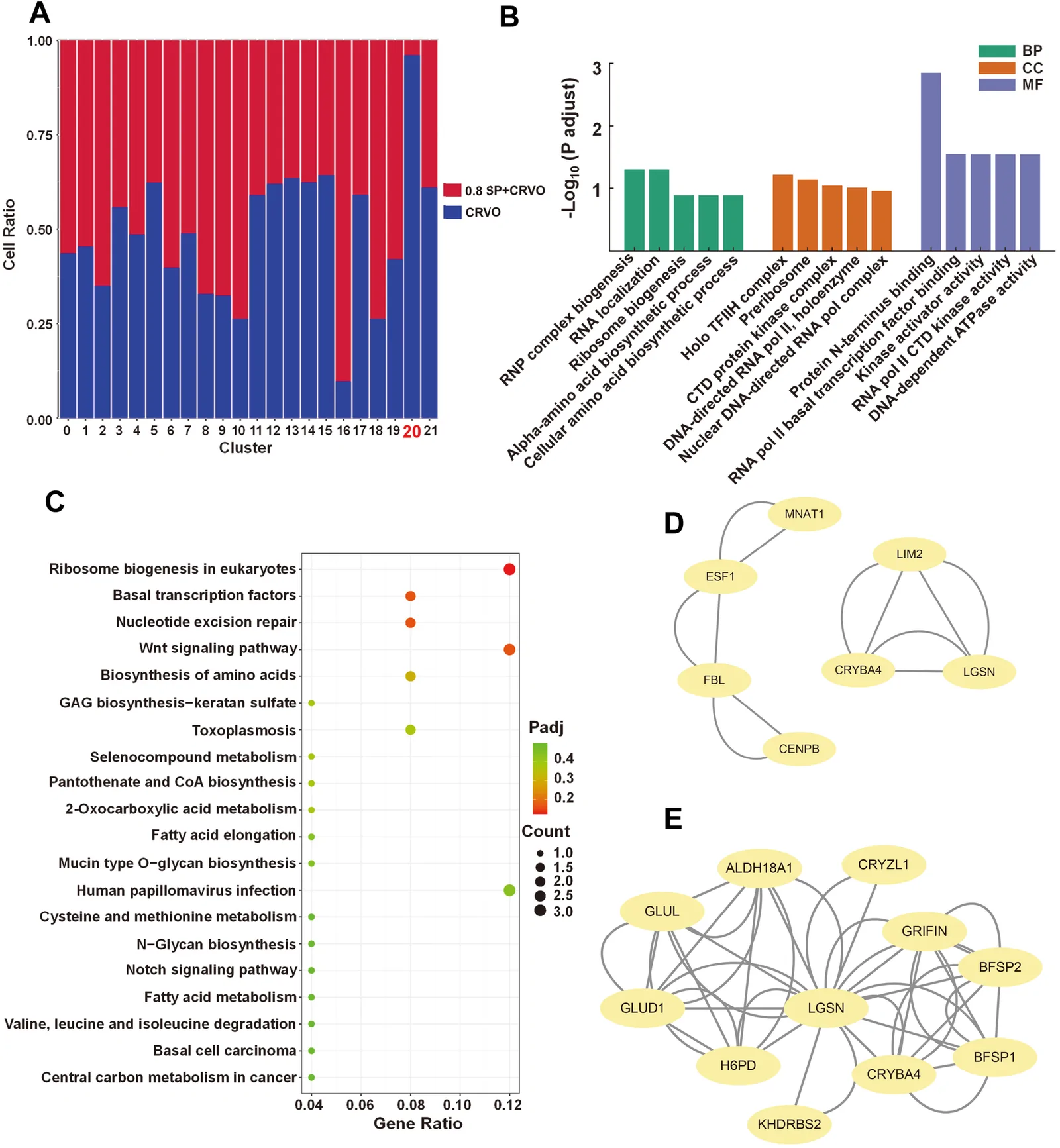
Characterization of cell cluster 20. **(A)** The proportions of the 22 identified cell clusters in the 2 experimental groups. **(B)** GO enrichment analysis of the DEGs in cell cluster 20. **(C)** KEGG enrichment analysis of the DEGs in cell cluster 20. **(D)** The PPI network of the DEGs in cell cluster 20. **(E)** The PPI network of the LGSN.

The proportions of the identified cell clusters from the 2 groups were quantified. Notably, the proportion of Müller cells in the 0.8 SP + CRVO group decreased (Figure 7A). In particular, cluster 20, which represented a subpopulation of Müller cells, exhibited the most dramatic reduction both within the Müller cell population and among the 22 identified cell clusters (Figure 7A). In contrast, the proportions of several clusters of microglia, including clusters 0, 1, and 10, were greater in the 0.8 SP + CRVO group than in the CRVO group, whereas other clusters, such as clusters 4 and 7, remained largely unchanged (Figure 7A). These findings indicate that microglial clusters 0, 1, and 10 may undergo reactive activation upon SP stimulation. Moreover, the number of cells in clusters 16 and 18, presumably two types of immune cells involved in retinal immune responses and inflammation, markedly increased in the 0.8 SP + CRVO group. Additionally, substantial decreases in the proportions of bipolar cells, cone photoreceptors, and endothelial cells were detected in the 0.8 SP + CRVO group (Figure 7A), implicating severe damage to these cell types in CRVO retinas exposed to high-dose SP.

#### 3.7.3 *Lgsn* overexpression may be associated with a drastic reduction in the number of a Müller cell cluster in CRVO retinas pretreated with high-dose SP

Cell cluster 20, defined as a subpopulation of Müller cells with high expression of marker genes such as *Dapl1*, *Slc1a3*, *Nnat*, and *Rlbp1* (Table 2), exhibited the most profound decline in cell proportion under high-dose SP stimulation (Figure 7A). By further comparing the gene expression profiles of cell cluster 20 between the CRVO and 0.8 SP + CRVO groups, a total of 58 DEGs were identified. Notably, compared with those in the CRVO group, the DEGs in the 0.8 SP + CRVO group were significantly upregulated, and no downregulated genes were detected. In particular, *Cryba4*, *Lgsn*, *Cenpb*, *Flot1*, *Trmt10c*, *Plcd3*, *Sephs1*, and *Nsa2* were the top 8 upregulated genes (Table 3).

**Table 3:** Top 8 upregulated genes in cluster 20 of CRVO retinas pretreated with SP.

Gene symbol	Gene name	Log**_2_** (Fold Change)	*P* adjust
*Cryba4*	Crystallin Beta A4	2.28	0.003
*Lgsn*	Lengsin	2.19	0.000
*Cenpb*	Centromere protein B	1.59	0.000
*Flot1*	Flotillin 1	1.26	0.000
*Trmt10c*	TRNA methyltransferase 10C	1.21	0.000
*Plcd3*	Phospholipase C Delta 3	1.17	0.013
*Sephs1*	Selenophosphate synthetase 1	1.12	0.013
*Nsa2*	Nop seven associated 2	1.06	0.005

GO analysis revealed that the DEGs in Müller cell cluster 20 were enriched mainly in molecular functions, including protein N-terminal binding, RNA polymerase II basic transcription factor binding, kinase activator activity, RNA polymerase II C-terminal domain kinase activity, and DNA-dependent ATPase activity (Figure 7B). Moreover, the DEGs in cell cluster 20 were associated with two specific biological processes: RNA localization and ribonucleoprotein complex biogenesis (Figure 7B). KEGG analysis revealed that the DEGs were enriched in the pathways of eukaryotic ribosome biogenesis, basic transcription factors, nucleotide excision repair, and canonical Wnt signaling (Figure 7C).

The DEGs in cluster 20, which were related primarily to N-terminal protein binding, were significantly upregulated in Müller cells following SP stimulation. This upregulation likely intensifies the Müller cell damage associated with SP exposure. To gain further insight, the PPI network of the DEGs in cell cluster 20 was analyzed. The results revealed interactions among the protein products encoded by the *Lgsn*, *Lim2*, and *Cryba4* genes, as well as among the products of the genes *Mnat1*, *Esf1*, *Fbl*, and *Cenpb* (Figure 7D).

Among the proteins that potentially interact with LGSN, several candidates associated with lens function, including CRYBA4, BFSP1, and GRIFIN, have been identified. Moreover, proteins related to glutamine metabolism, such as GLUL, GLUD1, and ALDH18A1 (Figure 7E), were also identified, suggesting the association of Müller cell glutamine metabolism with aggravated lesions in SP-treated CRVO retinas.

### 3.8 IF staining validated LGSN upregulation in Müller cells in SP-pretreated CRVO retinas

**Figure 8:**
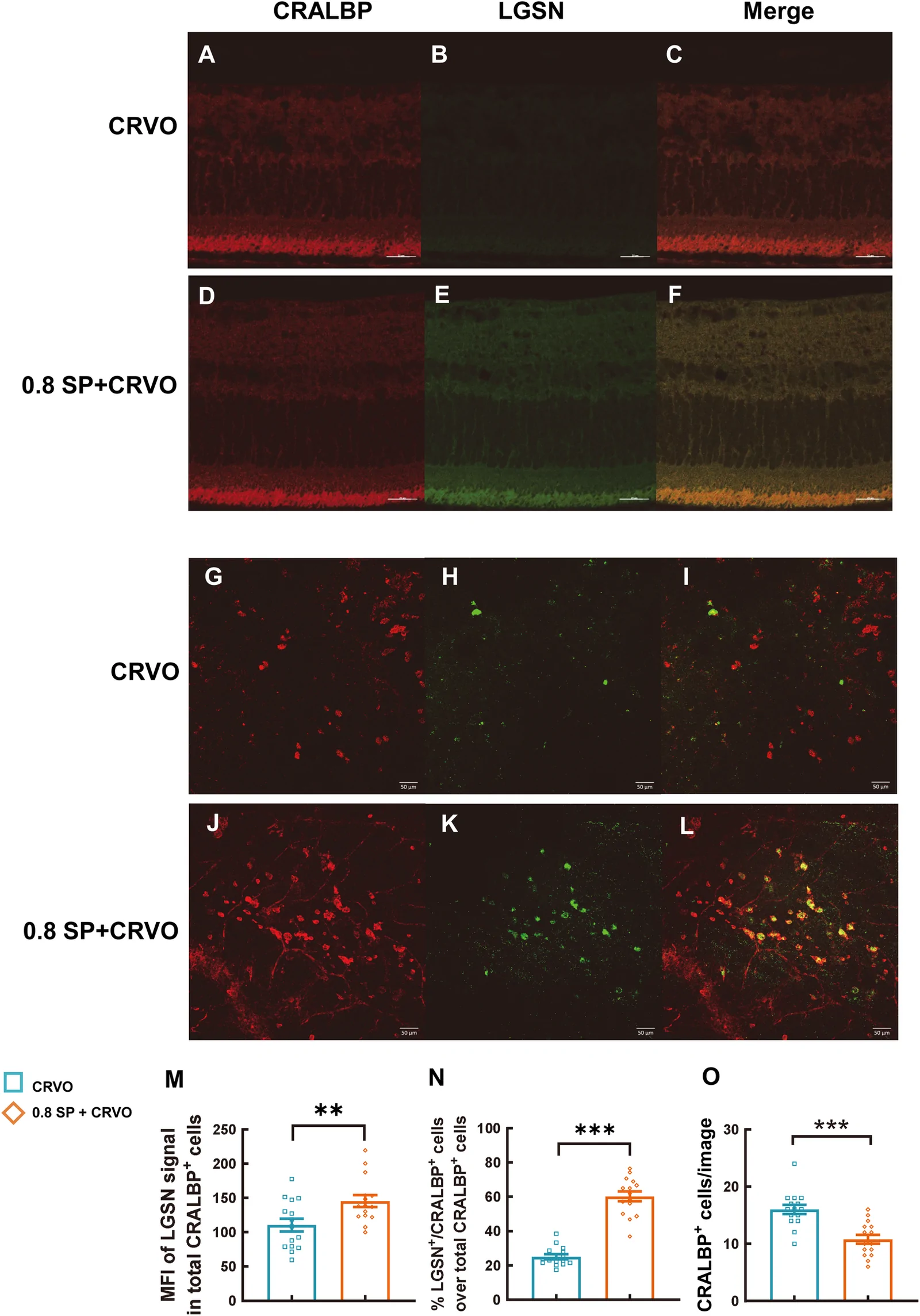
IF validation of LGSN expression in retinal Müller cells. **(A,D)** IF staining of CRALBP in the CRVO and 0.8 SP + CRVO groups of retina paraffin sections. **(B,E)** IF staining of LGSN in the CRVO and 0.8 SP + CRVO groups of retina paraffin sections. **(C,F)** Merged images of paraffin section staining. Scale bar: 20 µm. **(G,J)** IF staining of CRALBP in CRVO and 0.8 SP + CRVO retina whole mounts. **(H,K)** IF staining of LGSN in CRVO and 0.8 SP + CRVO retina whole mounts. **(I,L)** Merged images of retina whole mount staining. Scale bar: 50 µm. *n* = 5. **(M)** The MFI of the LGSN signal in total CRALBP^+^ cells. **(*N*)** The percentage of LGSN^+^ and CRALBP^+^ cells over total CRALBP^+^ cells. **(O)** The number of CRALBP^+^ cells per image. *n* = 15 images/group. The data are presented as the means ± SEMs. **p < 0.01, ***p < 0.001; unpaired t test.

In paraffin sections, IF signals of cellular retinaldehyde-binding protein (CRALBP, encoded by the *Rlbp1* gene), a major marker of cell cluster 20, were detected throughout the retinal layers, exhibiting the typical morphology of retinal Müller cells (Figures 8A,D). The staining intensity of CRALBP in the CRVO retinal sections was higher than that in the 0.8 SP + CRVO retinal sections (Figures 8A,D), which is presumably due to the reduction in the number of Müller cells in the SP-pretreated CRVO retinas. Notably, LGSN staining was low in the CRVO retinal sections but markedly increased in the 0.8 SP + CRVO sections (Figures 8B,E). The IF staining of LGSN was mostly colocalized with that of CRALBP (Figures 8C,F).

The IF staining patterns in paraffin sections were validated in whole mount retinas. The LGSN staining was weak in the CRVO retinas (Figure 8H). However, following SP treatment, both the MFI and the positive percentage of LGSN signal in total CRALBP^+^ cells (Müller cells) were dramatically increased (Figures 8K–M, both *p* < 0.01, CRVO vs. 0.8 SP + CRVO), and a significant reduction in the number of CRALBP^+^ cells (Müller cells) was also detected (Figures 8G–N, *p* < 0.001, CRVO vs. 0.8 SP + CRVO).

Taken together, these results were consistent with the upregulated expression of the *Lgsn* gene in a subpopulation of Müller cells with diminished cell number (cluster 20), as revealed by scRNA sequencing (Tables 2, 3).

### 3.9 Gene expression and functional validation in human Müller cell cultures

**Figure 9:**
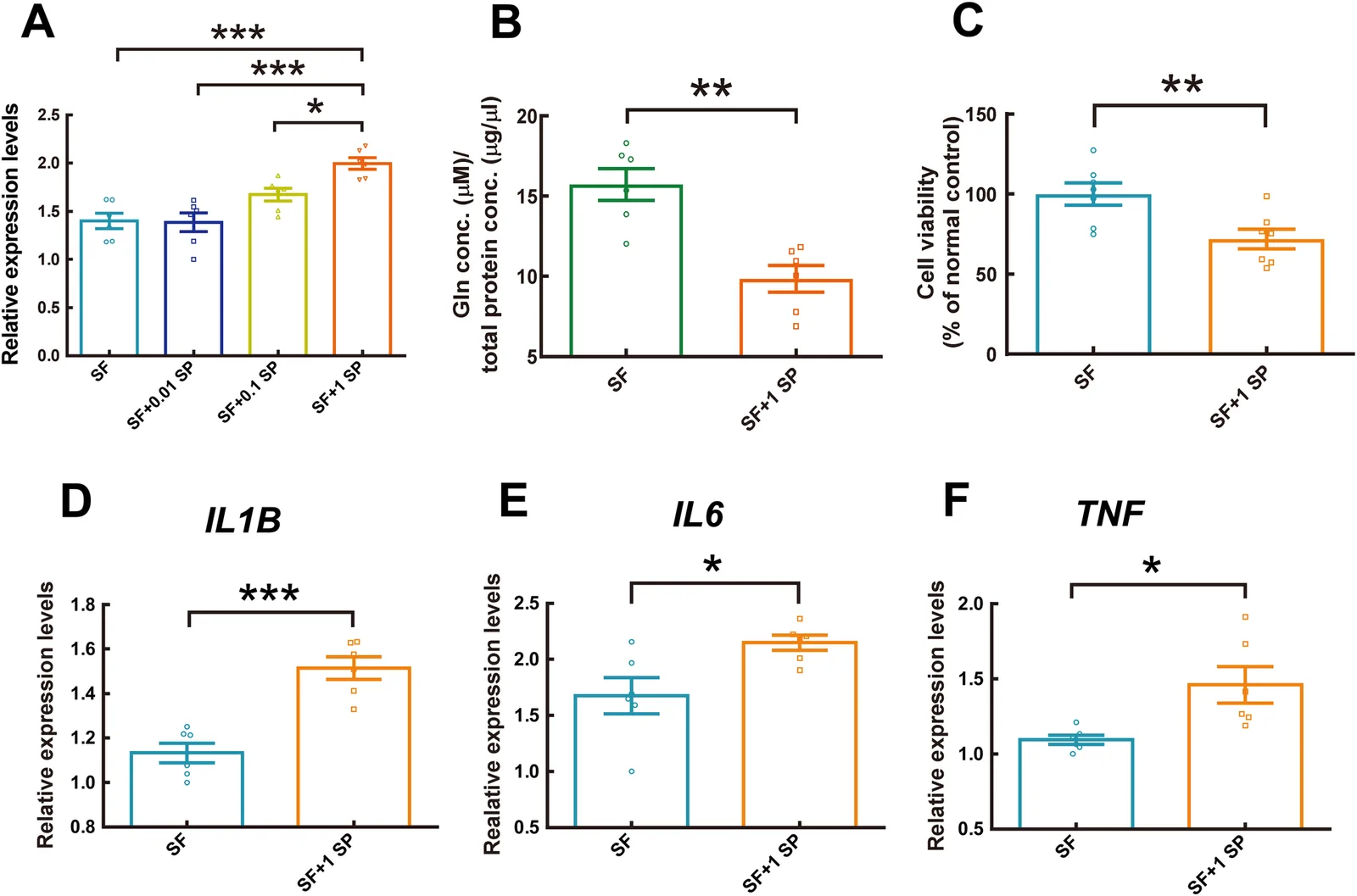
Effects of SP on human Müller cell cultures. **(A)** SP at 0.01, 0.1, and 1 μg/mL dose-dependently upregulated *LGSN* gene expression. The effects of 1 μg/mL SP on intracellular glutamine levels **(B)**, cell viability **(C)**, and the expression of the genes encoding *IL1B* **(D)**, *IL6* **(E)**, and *TNF* **(F)** *n* = 6–7. The data are presented as the means ± SEMs. *p < 0.05, **p < 0.01, ***p < 0.001; one-way ANOVA followed by Tukey's *post hoc* test or unpaired t test.

MIO-M1, a human immortalized retinal Müller cell line, highly expresses the marker genes *RLBP1* and *SLC1A3* (43, 44) in the cytoplasm, as well as an inducible level of ACE2, the SARS-CoV-2 entry receptor, on the cell membrane (45). Therefore, the MIO-M1 cells were suitable for *in vitro* validation of the changes in Müller cell gene expression profiles and functions detected in the SP + CRVO mouse model. SF culture media were used to mimic the ischemic and hypoxic microenvironment of the CRVO retina. Under SF conditions, recombinant SP significantly upregulated the expression of the *LGSN* gene in a dose-dependent manner, with SP at 1 μg/mL exerting the most robust effect (Figure 9A; both *p* < 0.001, SF vs. SF + 1 SP, SF + 0.01 SP vs. SF + 1 SP; *p* < 0.05, SF + 0.1 SP vs. SF + 1 SP). Moreover, SP at the highest dosage reduced the intracellular glutamine level by 37.44% (Figure 9B; *p* < 0.01, SF vs. SF + 1 SP) and cell viability by 28.09% (Figure 9C; *p* < 0.01, SF vs. SF + 1 SP). Additionally, SP treatment also significantly upregulated the expression of proinflammatory factor genes, including interleukin 1*β* (*IL1B*), interleukin 6 (*IL6*), and tumor necrosis factor (*TNF*) (Figures 9D–F; SF vs. SF + 1 SP, *p* < 0.001, for *IL1B*; both *p* < 0.05, for *IL6* and *TNF*). These results indicated that direct SP exposure to retinal Müller cells was associated with the upregulation of *LGSN* and proinflammatory factor genes as well as attenuated glutamine metabolism and cell viability.

## 4 Discussion

The results of this study suggested that intravitreal exposure to high-dose full-length recombinant SP, the major pathogenic factor of SARS-CoV-2, was associated with aggravated retinal lesions, including delayed reperfusion of occluded vessels, exacerbated vascular leakage and retinal edema, and impaired retinal electrophysiological functions, in a mouse model of laser-induced CRVO (Figures 2–5). Furthermore, scRNA sequencing revealed dramatic upregulation of the *Lgsn* gene in a subpopulation of retinal Müller cells in the CRVO retinas pretreated with high-dose SP (Figures 6, 7), which was validated in paraffin sections and whole mount retinas of the mouse model (Figure 8) and a culture paradigm of human retinal Müller cells (Figure 9A). In addition, functional consequences of SP stimulation, such as deterioration in glutamine metabolism and cell viability and upregulation of proinflammatory factor genes, were explored in the human Müller cells. This study provided clues regarding retinal vascular and edematous lesions and functional deterioration associated with intravitreal SP exposure and might shed light on potential cellular and molecular targets for COVID-19 patients with comorbidity of CRVO.

In the clinical setting, the main causes of CRVO include but are not limited to endothelial damage to retinal vessels, a hypercoagulable state, and hemodynamic changes, resulting in retinal vein thrombosis (46, 47). The obstruction of blood flow in the veins leads to insufficient perfusion, ischemia, and hypoxia in the retina. As a result, the levels of cytokines such as IL6, interleukin 8, monocyte chemoattractant protein-1, and vascular endothelial growth factor are significantly elevated, causing sustained damage to the BRB, vascular leakage, and retinal edema (48). In this study, retinal veins were experimentally occluded using laser photocoagulation after intravenous injection of Rose Bengal, a photoactivator dye that promotes thrombus formation. During the early phase of the CRVO model, blocked blood flow and severe impairment of the BRB recapitulated retinal edema. Reperfusion of the occluded vessels and the formation of collateral vessels subsequently increased vascular perfusion in the retina (Figure 2). During the late phase of the CRVO model, the edema subsided, but retinal atrophy ensued (Figure 3). Moreover, when the vascular lesions peaked, the electrophysiological functions of CRVO retinas were also severely compromised, as demonstrated by prolonged implicit time, subdued amplitudes, and shrunken b/a ratio in ERGs (Figures 4, 5). The course and phenotypes of the CRVO model in the current study were consistent with those in the literature (49, 50).

This model was subsequently used to explore the association of intravitreal SP exposure with the severity of retinal lesions in CRVO. SP is the major pathogenic factor of SARS-CoV-2 and is localized in the protein shell of this virus. The SP binds to ACE2 on human cells to facilitate viral infection and initiate pathological cascades. Upon attachment to the cell membrane, the SP is cleaved by host proteases into S1 and S2 subunits. The S1 subunit contains an N-terminal domain and a C-terminal domain, both of which act as receptor-binding domains (RBDs). Although the SPs of early SARS-CoV-2 strains bind to ACE2 in hamsters, ferrets, and nonhuman primates, they do not bind to ACE2 in mice (51). In contrast, the SP of the B.1.1.529 Omicron variant has 30 amino acid substitutions, 3 deletions, and 1 insertion (52). Modeling data predict that the SP of the Omicron variant binds to mouse ACE2 through the substitutions N501Y and Q493R in the RBD. This prediction has been verified by the symptoms of viral transmission and replication in the respiratory tract in mice infected with the Omicron variant (53, 54), and the eye has been considered a potal of entry, a reservior of replication, and a source of contagion for the virus (55). Therefore, the recombinant SP of the B.1.1.529 Omicron variant was used in the current study for intravitreal injection 2 days prior to CRVO induction. As a result, the venous reperfusion rates of the CRVO retinas pretreated with various concentrations of SP were lower than those of the CRVO retinas without SP pretreatment; consistently, the intravascular intensities of the fluorescence dye in the retinas of the SP + CRVO groups were also lower than those in the CRVO group, although no statistically significant differences in these two proxies were detected between the groups (Figures 2A–I). Moreover, the breakdown of the BRB and retinal edema were substantially aggravated in the group pretreated with the high concentration of SP, as indicated by the significantly expanded area of the extravascular fluorescence dye in the FFA (Figures 2A,N–Q) and the significantly increased thickness of the total retina in the OCT (Figure 3). In the CRVO mice pretreated with high-dose SP, the significant exacerbation of phenotypes was maintained throughout the experiment and peaked on the 9th day after laser photocoagulation (Figures 2–5). Therefore, the retinas from the CRVO group and the 0.8 SP + CRVO group on the 9th day after modeling were analyzed by scRNA sequencing to explore the cellular and molecular clues associated with the exacerbation.

ScRNA sequencing revealed 22 cell clusters; however, 2 of them remained undefined (Supplementary Figure 3). One is cluster 16. Among its highly expressed genes, S100A8/S100A9 is the most abundant cytoplasmic protein complex expressed in mature neutrophils; however, this complex is also a hallmark of activated inflammatory monocytes/macrophages (56). Moreover, CXCL2 is prominently produced by neutrophils and inflammatory macrophages for the recruitment of sufficient circulating leukocytes (57). In addition, SRGN is highly expressed in neutrophil granules and myeloid innate immune cells (58), where it regulates cell adhesion and migration and granule cargo storage (Supplementary Figure 3A). On the other hand, the results of the GO analysis showed that the highly expressed genes were enriched in inflammatory response, cytokine synthesis, and cell adhesion processes (Supplementary Figure 3B), which are associated with the core functions of neutrophils, such as transendothelial migration via adhesion molecules, cytoskeleton remodeling, and proinflammatory mediator release. Therefore, this cluster of cells could be composed of neutrophils that massively infiltrated the retina during the early phase of CRVO and SP exposure. Alternatively, in view of the fact that scRNA sequencing was performed at 9 days following CRVO induction, this cell cluster could also be composed of inflammatory monocyte-derived macrophages that were recruited from the periphery and accumulated in the retina at later phases of CRVO.

Another undefined cell cluster is cluster 18. Among the highly expressed genes, PTPRC is universally expressed on mature T lymphocytes and is essential for T cell activation (59, 60). GZMA is a cytotoxic marker of CD8⁺ effector T cells (61). CCL5 is enriched in activated effector/memory CD8⁺ or CD4^+^ T cells and is secreted upon T cell activation to drive intraretinal immune cell recruitment (62, 63). Furthermore, AW112010 is a mouse long noncoding RNA whose expression is specifically upregulated in activated inflammatory T helper 1/CD4^+^ effector T cells (64) (Supplementary Figure 3C). The expression patterns of the highly expressed genes were consistent with the results of the GO enrichment analysis. Ribosome biosynthesis and cytoplasmic translation were substantially promoted after T cell activation to support effector protein synthesis and clonal proliferation (Supplementary Figure 3D). Thus, the primary cell type candidate of cluster 18 could be infiltrated activated cytotoxic CD8⁺ effector T lymphocytes, with activated CD4⁺ effector T cells serving as an alternative.

More importantly, the results of scRNA sequencing revealed that compared with CRVO retinas without SP treatment, high-concentration SP-treated CRVO retinas exhibited the most dramatic reduction in the proportion of a subpopulation of Müller cells (Figure 7A). Indeed, Müller cells express ACE2 (45, 65), to which SP can directly bind and elicit deleterious cascades. In general, Müller cells are important components of the BRB and mediate neurovascular coupling and control retinal vascular activity (66); they also maintain the metabolic support of neurons, participate in ion transport, and regulate the microenvironment in the retina (67). More specifically, in the retina, Müller cells are involved in a unique metabolic cycle, the glutamate‒glutamine cycle. Glutamate, one of the most common excitatory neurotransmitters, is released by retinal neurons, transported into Müller cells via excitatory amino acid transporter 1, and converted into glutamine through glutamine synthetase (GS). Glutamine is then released by Müller cells and taken up and converted into glutamate by neurons to replenish the repertoire of the excitatory neurotransmitter (68). Thus, Müller cells play crucial roles in the clearance of extracellular glutamate and the synthesis of glutamine.

Furthermore, the results of the scRNA sequencing in this study revealed a 4.56-fold increase at the transcript level of the *Lgsn* gene in a subpopulation of Müller cells in the SP-stimulated CRVO retinas relative to that in the CRVO retinas (Table 3). Dose-dependent upregulated expression of the *LGSN* gene was validated in a human Müller cell line stimulated with different concentrations of SP (Figure 9A). Moreover, according to the data derived from a webtool (https://covid-omics.app) that was generated on the basis of multiomics analyses of blood samples from patients with or without COVID-19 (69), the transcript levels of *LGSN* in leucocytes from COVID-19 patients were significantly higher than those from patients without COVID-19, indicating the clinical relevance of these findings.

At the protein level, LGSN was low in the retinal sections and whole mount retinas of CRVO mice (Figures 8B,H), which was consistent with previous findings that the *Lgsn* gene is preferentially expressed in lens cells (70). Nevertheless, the abundance of its protein product greatly increased in Müller cells from CRVO retinas after treatment with 0.8 μg SP (Figures 8E,K–N), indicating that LGSN upregulation may be an ectopic overexpression that is associated with a special pathological condition, i.e., intravitreal SP exposure in the context of CRVO. Furthermore, structural studies have demonstrated that the LGSN, a class of crystallin with a GS domain, harbors most of the conserved sites for glutamate and Mg^2+^ binding (71). In contrast, major deletions have been detected at the catalytic sites of the GS domain in the LGSN (72), rendering this protein completely devoid of glutamine synthesis activity; thus, the LGSN is also known as pseudo-GS and may function as a molecular chaperone (70, 73). Although the PPI network analysis revealed several protein candidates involved in glutamine metabolism that potentially interact with LGSN (Figure 7E), the most straightforward speculation could be that the excessive LGSN might bind to intracellular glutamate but fail to synthesize glutamine, thereby disrupting the glutamate‒glutamine cycle in Müller cells in a dominant-negative manner. Although this speculation remains to be verified, the net effect is a reduction in intracellular glutamine levels, which has been validated in SP-stimulated human Müller cells (Figure 9B) in this study. Moreover, several clinical studies have consistently reported reduced glutamine levels in blood samples from COVID-19 patients (74–76). Furthermore, the reduced glutamine level was reported to be associated with a weakened capacity of Müller cells to respond to oxidative stress (77), congruent with the reduced viability of the SP-stimulated human Müller cells (Figure 9C) in the current study. The upregulation of proinflammatory factors in human Müller cells upon SP stimulation (Figures 9D–F) was consistent with the results from a transcriptomic analysis of tears from COVID-19 patients (78), indicating that a dysregulated immune response and inflammation may be involved in ocular infection with SARS-CoV-2.

There are several limitations in this study. First, the intravitreal administration of recombinant SP does not fully recapitulate the pathological process of SARS-CoV-2 infection; hence, experiments involving direct viral infection in animals are necessary to substantiate the translational value of the findings in this study. Second, pooling retinal samples for scRNA sequencing blunts the individual variability of biological samples and may compromise data reproducibility. Third, additional experiments are needed to corroborate the causal relationship between LGSN upregulation in a subpopulation of Müller cells and aggravated lesions in SP-treated CRVO retinas.

## 5 Conclusion

In summary, intravitreal exposure to full-length recombinant SP, the major pathogenic factor of SARS-CoV-2, was associated with aggravated retinal lesions, including delayed vascular reperfusion, exacerbated vascular leakage and retinal edema, and deteriorated electrophysiological functions, in a mouse model of laser-induced CRVO. Furthermore, scRNA sequencing of CRVO retinas treated with or without high-dose SP revealed dramatic upregulation of LGSN in a subpopulation of Müller cells, which was validated in both CRVO mouse retinas and SP-stimulated human Müller cell cultures. The findings in this study may provide clues on potential cellular and molecular targets for COVID-19 patients with comorbidity of CRVO.

## Data Availability

The original contributions presented in the study are publicly available. The data GSA: CRA032814 (title: Single-cell RNA sequencing analysis of the effects of the SARS-CoV-2 spike protein in a murine model of CRVO) have been released and made available to public. The data can be accessed and downloaded through the link https://ngdc.cncb.ac.cn/gsa/browse/CRA032814. Further inquiries can be directed to the corresponding author.
