# Supplementary material for: Intravitreal exposure to the SARS-CoV-2 spike protein is associated with aggravated retinal lesions in a murine model of central retinal vein occlusion
Source: Front Med (Lausanne). 2026 Sep 3;13:1794421. doi: 10.3389/fmed.2026.1794421 (PMC13581845; doi:10.3389/fmed.2026.1794421)
Supplement: Supplementary file 1 [file Supplementaryfile1.docx]

Supplementary Material

# 1 Supplementary Figures

**Supplementary Figure 1.** An illustration of retina OCT images. (A) The green lines represent 19 equidistant optical sections of the mouse retina, with the optic disc at the center. The line with an arrow head indicates the position of the current optical section. (B) The OCT image of the current section.

# Supplementary Figure 2. An illustration of built-in software for retinal thickness calculation in Heidelberg OCT machine. (A) The position of the ETDRS ring in a fundus image. The center of the ETDRS ring was placed at the optic disc of the mouse retina. (B) For each OCT image, Heidelberg OCT software measured the thickness from the Bruch’s membrane to the inner limiting membrane. (C) The scanning area did not include the outer circle of the ETDRS ring, therefore, only the thicknesses in the center and inner circle were measured, and the average value was calculated to represent the retinal thickness. ETDRS: Early Treatment Diabetic Retinopathy Study.

# Supplementary Figure 3. Characterization of undefined cell clusters. (A) The expression of top 4 highly expressed genes in cell cluster 16. (B) The GO analyses of highly expressed genes in cell clusters 16. (C) The expression of top 4 highly expressed genes in cell cluster 18. (D) The GO analyses of highly expressed genes in cell clusters 18.
